# Blocking apoptosis promotes survival and alters developmental dynamics of human retinal ganglion cells in retinal organoids

**DOI:** 10.1016/j.celrep.2026.117270

**Published:** 2026-04-16

**Authors:** Jingliang Simon Zhang, Brian Guy, Clayton P. Santiago, Caterina Tiozzo, Meghana Sreenath, Ya-Wen Chen, Seth Blackshaw, Robert J. Johnston

**Affiliations:** 1 Department of Biology, Johns Hopkins University, 3400 N. Charles Street, Baltimore, MD 21218, USA; 2 The Solomon H. Snyder Department of Neuroscience, Johns Hopkins Medical Institute, Baltimore, MD 21218, USA; 3 Division of Neonatology, Department of Pediatrics, Icahn School of Medicine at Mount Sinai, New York, NY 10029, USA; 4 Department of Otolaryngology, Icahn School of Medicine at Mount Sinai, New York, NY 10029, USA; 5 Department of Cell, Developmental, and Regenerative Biology, Icahn School of Medicine at Mount Sinai, New York, NY 10029, USA; 6 Institute for Airway Sciences, Icahn School of Medicine at Mount Sinai, New York, NY 10029, USA; 7 Institute for Regenerative Medicine, Icahn School of Medicine at Mount Sinai, New York, NY 10029, USA; 8 Lead contact

## Abstract

Retinal ganglion cells (RGCs) are the projection neurons connecting the retina to the brain. In many species, a substantial proportion of RGCs are eliminated by programmed cell death during development to regulate their final number, but how cell death impacts human RGC development remains poorly understood. Here, we characterized cell death in human fetal retinas and retinal organoids. Both retinas and organoids exhibited two waves of apoptosis: an early wave targeting neurogenic retinal progenitor cells and neuronal precursors and a late wave affecting RGCs and other neurons. Additionally, organoids displayed a distinct wave of necrosis. Blocking apoptosis in organoids via *BAX*/*BAK* double knockout improved RGC survival but delayed RGC neurogenesis and maturation. Our results highlight the roles of apoptosis in human RGC development and the challenges in retinal organoid design. Addressing these limitations will improve the utility of organoids for studying human retinal development and modeling optic neuropathies such as glaucoma.

## In brief

Zhang et al. found that two waves of apoptosis occur in developing human retinas and retinal organoids, affecting RPCs and RGCs, respectively. Retinal organoids undergo an additional wave of necrosis in the core, further eliminating RGCs. Blocking apoptosis in organoids promotes RGC long-term survival and delays their neurogenesis and maturation.

## Graphical Abstract

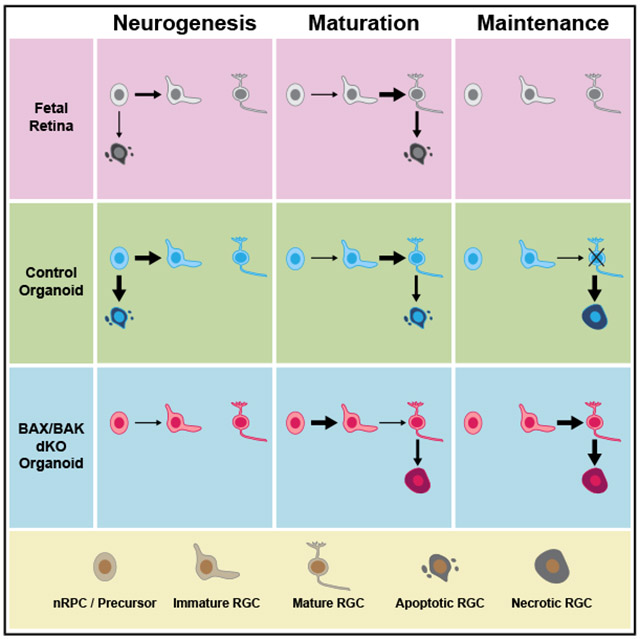

## INTRODUCTION

The human retina is a laminated structure in the eye that enables vision. In the adult human retina, cell bodies of retinal neurons are distributed in three nuclear layers: the outer nuclear layer (ONL), the inner nuclear layer (INL), and the ganglion cell layer (GCL).^1,2^ Located in the GCL, retinal ganglion cells (RGCs) are the output neurons of the retina, projecting axons through the optic nerve to transmit visual information to the brain.^3^ As the first neuronal cell type specified in the human retina,^4^ RGCs are overproduced during early development.^5^ Whereas the majority of RGCs die later in development, a subset of RGCs survive, establishing their topography across the retina and stable retinotopy to the brain.^6,7^ Here, we study how programmed cell death regulates human RGC neurogenesis, maturation, and survival.

Programmed cell death is a conserved mechanism that regulates cell number and refines neuronal connectivity in the retina and other parts of the central nervous system.^8-11^ Two distinct waves of programmed cell death occur in the developing retina of many species.^12-18^ The first wave occurs in the neuroblastic layer (NBL) and coincides with RGC neurogenesis.^17-19^ The second wave occurs during RGC innervation and maturation, eliminating RGCs that fail to innervate their targets correctly.^20-24^ Inhibition of apoptosis in the developing retina results in reduced developmental cell death, thickened retinal layers, increased RGC numbers, and impaired retinal functions.^17,25-28^ Thus, developmental apoptosis regulates RGC population size to ensure retinal function.

Our understanding of how cell death occurs and regulates human retinal development is limited. Human RGC neurogenesis starts in fetal week 6 and likely concludes by week 14, based on the temporal expression of key RGC developmental genes such as *ATOH7* and *POU4F2*.^29,30^ Caspase-3 (Casp3)-expressing cells are observed in human retinas between weeks 5 and 8, suggesting an early wave of apoptosis.^31^ A second apoptotic wave occurs later, with a peak between gestation weeks 16 and 20 (post-conception weeks [PCW] 14–18), when substantial loss of optic nerve axons and pyknosis of cells in the GCL are observed.^5,7,32,33^ This coincides with the stage when RGCs are sending axons to innervate their targets in the brain,^34-37^ suggesting that this second wave of cell death may refine the eye-to-brain connectivity by eliminating excess RGCs. However, the precise identity of apoptotic cells and the specific roles of cell death in human RGC development have not been studied.

**Figure 1.**
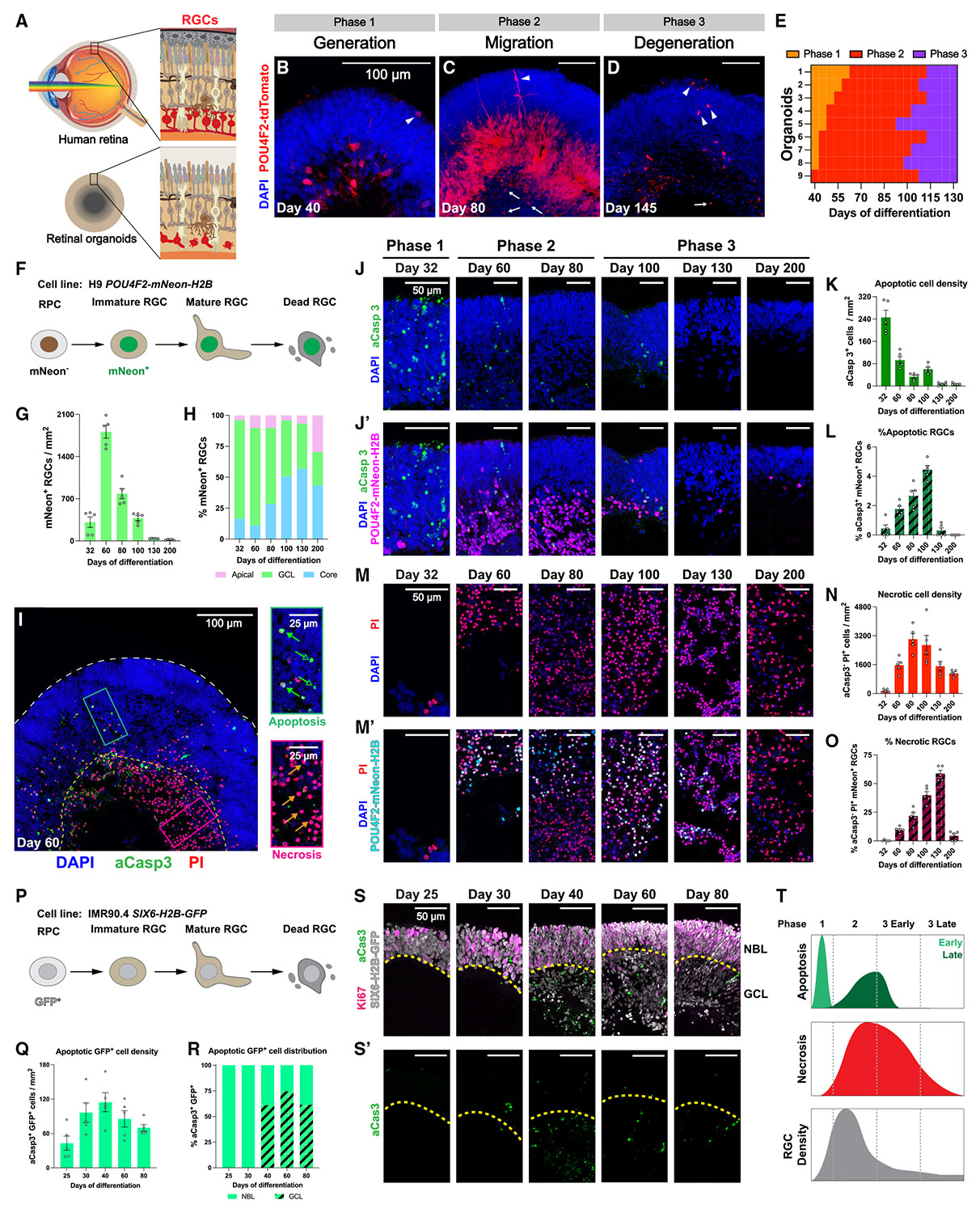
Waves of cell death in human retinal organoids (A) Schematic of RGCs (red) in the human retina and their loss in human retinal organoids. (B–D) Human retinal organoids in phase 1 (B), phase 2 (C), and phase 3 (D) with distinct abundances and distributions of tdTomato^+^ RGCs (red). White arrowheads indicate mislocalized RGCs in the apical layers. White arrows indicate mislocalized RGCs in the core. Scale bar, 100 μm. (E) Progression of RGC developmental phases in human retinal organoids. (F) *POU4F2-p2A-mNeon-H2B* labels immature, mature, and dead RGCs but not RPCs, as *POU4F2* is expressed in postmitotic RGC precursors. (G) Density of mNeon^+^ RGCs in human retinal organoids. Data are represented as the mean ± SEM. *n* = 5 organoids per time point. (H) Distribution of mNeon^+^ RGCs in different layers or regions of human retinal organoids. *n* = 5 organoids per time point. (I) Co-detection of apoptosis and necrosis in H9 *POU4F2-mNeon-H2B* retinal organoids. Empty green arrows indicate aCasp3^+^ PI^−^ cells (early apoptotic cells) and solid green arrows indicate aCasp3^+^ PI^+^ cells (late apoptotic cells) in retinal layers. Orange arrows indicate aCasp3^+^ PI^−^ cells (necrotic cells) in the core. Scale bar, 100 μm (left) and 25 μm (right). (J) Apoptotic cells (aCasp3^+^) and apoptotic RGCs (aCasp3^+^ mNeon^+^) in retinal layers. Scale bar, 50 μm. (K and L) Density of aCasp3^+^ cells in retinal layers (K) and percentage of aCasp3^+^ mNeon^+^ RGCs of all mNeon^+^ RGCs (L). Data are represented as the mean ± SEM. *n* = 5 organoids per time point. (M) Necrotic cells (PI^+^) and necrotic RGCs (PI^+^ mNeon^+^) in the core. Scale bar, 50 μm. (N and O) Density of aCasp3^−^ PI^+^ cells in the core (N) and percentage of aCasp3^−^ PI^+^ mNeon^+^ RGCs of all mNeon^+^ RGCs (O). Data are represented as the mean ± SEM. *n* = 5 organoids per time point. (P) SIX6-p2A-H2B-GFP labels RPCs, as well as immature, mature, and dead RGCs. (Q and R) Density of aCasp3^+^ GFP^+^ cells in retinal layers (Q) and distribution of aCasp3^+^ GFP^+^ cells in the NBL and GCL (R) during the early apoptotic wave. In (Q), data are represented as the mean ± SEM. *n* = 4 organoids per time point. (S) Detection of retinal cells (GFP^+^) and apoptotic retinal cells (aCasp3^+^ GFP^+^) during the early wave of apoptosis. The yellow dashed line indicates the boundary of the NBL and GCL based on Ki67 (magenta) expression in the NBL. Scale bar, 50 μm. (T) Schematic of RGC loss and waves of apoptosis and necrosis during human retinal organoid differentiation.

To address these questions, we studied cell death during the development of human fetal retinas and retinal organoids. Human stem cell-derived retinal organoids form optic vesicle-like structures (OVs) via evagination, generate all major neuronal cell types in the retina, and recapitulate early human retinal development *in vitro*, providing a highly accessible and experimentally tractable system for developmental and translational studies.^38-41^ In organoids, RGCs are generated in the inner layers and display neurite outgrowth, mimicking *in vivo* early RGC development.^42,43^ However, RGCs in organoids are progressively lost during long-term culture, compromising studies of RGC biology and their potential for translational applications (Figure 1A).^43,44^ It has been suggested that RGCs are lost in organoids due to the lack of innervation targets, neurotrophic support, vasculature, and/or inaccessibility of oxygen in the innermost region.^45-48^ Nevertheless, what drives RGC loss and how inhibition of apoptosis affects RGC development and maintenance in human organoids have not been examined.

Here, we investigated the spatiotemporal dynamics and functional roles of cell death in human fetal retinas and retinal organoids using immunofluorescence microscopy, live imaging, birth dating, and single-nucleus RNA sequencing (snRNA-seq). Our study describes the temporal waves and regulatory roles of cell death during human retinal development, providing a foundation for understanding the intricate mechanisms that shape retinal cell populations and advancing organoid model design to study neurodegenerative diseases.

## RESULTS

### Two waves of developmental apoptosis in the human fetal retina

Given the challenges in acquiring and analyzing fetal human retinal samples, previous analyses of retinal apoptosis were limited to time windows that encompassed only a single wave of developmental apoptosis and relied on morphological features rather than cell-type-specific markers to determine the identity of apoptotic cells.^5,7,31^

To evaluate the timing of the two waves of apoptosis and the identities of apoptotic cells during human retinal development, we evaluated postmortem human fetal retinas collected at PCW 9, 13, 16, and 20 using parasagittal sections (Figures S1A-S1D). We evaluated 3 retinas for PCW 9 and 2 retinas each for PCW 13, 16, and 20 from different individuals. Apoptotic cells were detected by immunofluorescent staining for active Casp3 (aCasp3), the cleaved form of Casp3 generated during apoptosis (Figures S1A′-S1D′).^49^ To identify apoptotic RGCs from other apoptotic cells in the GCL (e.g., starburst amacrine cells [SACs]),^50^ we co-stained with RNA binding protein with multiple splicing (RBPMS), an RGC-specific marker in the mammalian retina (Figures S1A-S1D).^51,52^

In the NBL, aCasp3^+^ cells were primarily observed at PCW 9 (1.4 ± 0.2 cells/mm) and decreased by PCW 13 (0.2 ± 0.1 cells/mm) (Figures S1A, S1B, S1E, and S1F), reminiscent of the first wave of developmental apoptosis affecting retinal progenitor cells (RPCs) and neuronal precursors in other vertebrate species.^17,19,53^

In the GCL, the density of apoptotic cells was low at PCW 9 (0.7 ± 0.2 cells/mm), increased by PCW 16 (1.6 ± 0.6 cells/mm), and then decreased by PCW 20 (0.2 ± 0.1 cells/mm) (Figures S1E and S1F), marking the second wave of developmental apoptosis in human fetal retina. Importantly, we observed apoptotic cells in the GCL that expressed RBPMS (71 out of 25,421 RBPMS^+^ RGCs examined at PCW 13 and 142 out of 16,449 RBPMS^+^ RGCs examined at PCW 16) (Figures S1B′, S1C′, S1G, and S1H), providing direct evidence that RGCs are the major cell type undergoing apoptosis in GCL during development.

To evaluate the spatial dynamics of developmental apoptosis in the human fetal retinas, we quantified the density of apoptotic cells and their distribution along the vertical meridian (Figures S1I-S1K). We found a high density of apoptotic cells in the NBL at PCW 9, which decreased by PCW 13 as the fetal retina grew in length (Figure S1I). We also observed high densities of apoptotic RGCs at PCW 13 and 16 (Figure S1J), followed by the establishment of a central peak of RGC density at PCW 20 (Figure S1K), consistent with analysis of horizontal sections of developing human retinas.^7,54^ In addition, we detected apoptotic cells in the INL, supporting previous reports of INL cell death during the second wave of apoptosis (Figures S1C′, S1E, and S1F).^17,33,55,56^

Together, these findings suggest that two waves of developmental apoptosis, an early wave affecting cells in the NBL and a later wave involving RGCs and other neurons, contribute to the sculpting of neuronal topography during human retinal development.

### Three phases of RGC development and loss in human retinal organoids

Human retinal organoids provide a highly accessible and experimentally tractable model to study human RGC development.^42,45,57^ To track changes in the spatial distributions and morphologies of RGCs during organoid development, we differentiated human retinal organoids from embryonic stem cells carrying the RGC-specific transgenic reporter *POU4F2-p2A-tdTomato* (H7 *POU4F2-tdTomato*)^58^ using a gravity aggregation-based protocol^59,60^ (Figure S2). After 25 days of differentiation, a subset of retinal organoids formed OVs, the evaginating immature retinal tissue (Figure S3A). By day 40, OVs were excised from the non-retinal parts and maintained in culture until day 200, while organoids lacking OVs were culled. Most OVs formed laminated retinal layers, including the GCL on the basal side, other retinal layers on the apical side (NBL between days 25 and 100 or ONL and INL between days 100 and 200), and a core with low cell density in the innermost region during culture (Figure S3B).

Our observations revealed three phases of RGC development and loss in retinal organoids (Figures 1B-1D): generation (phase 1), migration (phase 2), and degeneration (phase 3). These phases are distinct from the three general stages of human retinal organoid development previously characterized based on organoid morphology.^43^

In phase 1 organoids, most tdTomato^+^ RGCs were in the putative GCL at the basal side of the retinal layers (Figure 1B). In phase 2 organoids, tdTomato^+^ RGCs started extending neurites. During this phase, although most RGCs still remained in the GCL, a subset of RGCs were mislocalized along extending neurites in the apical NBL or in the core (Figure 1C), suggesting migration from the GCL. Also, pyknotic nuclei accumulated in the core during phase 2 (Figure 1C). In phase 3 organoids, fewer tdTomato^+^ RGCs were observed. RGCs appeared to retract their neurites and retain fluorescent signal mainly in their soma (Figure 1D), indicative of degeneration. The progressive loss of tdTomato^+^ RGCs in human retinal organoids aligns with previous studies using different methods of organoid differentiation,^43,45^ suggesting that RGC loss is a common feature of long-term retinal organoid culture.

To determine when retinal organoids progress through each phase of RGC development, we tracked the development of tdTomato^+^ RGCs in nine individual retinal organoids from day 40 to 130 (Figures 1E and S3C). While organoids began the observation period in different phases and the absolute timing of each phase varied, the relative timing and progression were consistent across all organoids (Figure 1E). Phase 1 was relatively short, with a maximum duration of 25 days from the start of observation. Phase 2 was observed primarily between days 50 and 100. All organoids entered phase 3 by day 110 and remained in this phase (Figure 1E).

Taken together, these results demonstrate that RGCs in organoids develop from phase 1 (generation, days 25–50) to phase 2 (migration, days 50–100) and, finally, to phase 3 (degeneration, days 100–200) (Figures S3D-S3G), providing a general timeline for RGC development and loss in human retinal organoids.

### Tracking RGCs with a permanent transgenic reporter in retinal organoids

The progressive loss of *POU4F2-p2A-tdTomato*-expressing human RGCs could be due to downregulation of *POU4F2* during normal RGC maturation^61,62^ or cell death.^63^ To track RGCs throughout their lifespan, we differentiated human retinal organoids from embryonic stem cells carrying a stable transgenic RGC-specific nuclear reporter, *POU4F2-p2A-mNeon-H2B* (H9 *POU4F2-mNeon-H2B*).^64^ Because *POU4F2* is expressed in postmitotic immature RGCs and fluorescent proteins fused with histone proteins remain stably associated with chromatin in postmitotic neurons,^65-67^ this reporter permanently labels RGCs that express *POU4F2* as they mature or undergo cell death (Figures 1F and S3H). Moreover, the nuclear mNeon reporter facilitates more accurate quantification of RGCs in organoids by minimizing confounding signals from overlapping RGC soma and neurites with the cytosolic tdTomato reporter (Figure 1C).

To evaluate the dynamics of mNeon^+^ RGC abundance, we quantified the density of mNeon^+^ RGCs in organoids on days 32, 60, 80, 100, 145, and 200. The density was low on day 32 in phase 1, peaked on day 60 in early phase 2, and progressively declined from day 80 onwards in late phase 2 and phase 3 (Figure 1G). The loss of permanently labeled mNeon^+^ RGCs directly demonstrates RGC death and clearance during organoid culture, consistent with our previous observation in H7 *POU4F2-tdTomato* organoids (Figures 1B-1D).

To assess the dynamics of mNeon^+^ RGC migration, we quantified the percentage of mNeon^+^ RGCs in the GCL, apical layers (NBL or ONL+INL), and core. In phase 1 on day 32, most mNeon^+^ RGCs were located in the GCL (Figure 1H). In phase 2 on day 60, the proportion of mNeon^+^ RGCs in the GCL decreased, while the proportions in the apical layers and core increased (Figure 1H), consistent with RGC migration from the GCL (Figure 1C). In phase 3, from day 100 on, the proportion of mNeon^+^ RGCs in the GCL continued to decline, while the proportion in the core increased between days 100 and 130 and then decreased by day 200 (Figure 1H), suggesting distinct dynamics of RGC loss in the GCL and the core. Together, these results define the spatiotemporal dynamics of RGC neurogenesis, migration, and loss during human retinal organoid development.

### RGCs die by apoptosis and necrosis in retinal organoids

The loss of mNeon^+^ RGCs suggested that RGCs undergo cell death during long-term organoid culture. Apoptosis of RGCs occurs in developing human retinas (Figures S1B, S1C, and S1H)^5,7,32^ and mouse stem-cell-derived retinal organoids *in vitro*.^68^ In addition, the migration and loss of mNeon^+^ RGCs in the core of OVs (Figure 1H and S3H) is reminiscent of the accumulation of necrotic cells in the core of human brain organoid models.^69-71^ Thus, we hypothesized that RGC loss in human retinal organoids results from apoptosis and/or necrosis.

Propidium iodide (PI) is a cell-impermeable, red fluorescent nuclear dye that marks the nuclei of cells undergoing membrane rupture during necrosis and late-stage apoptosis.^72,73^ Co-detection with apoptosis-specific markers and PI is commonly used in flow cytometry to distinguish apoptotic and necrotic cells,^72,74,75^ but this approach has not been applied to immunofluorescent staining of organoid sections. To investigate the forms of cell death in human retinal organoids, we developed an imaging-based co-detection assay using the antibody against aCasp3 and the dye PI. While aCasp3 marks apoptotic cells, PI labels nuclei of late-stage apoptotic cells, necrotic cells, and cells dying via other mechanisms such as necroptosis^76^ or secondary necrosis due to the lack of phagocytes to clear apoptotic cells.^77^ Here, we classified aCasp3^+^ PI^−^ cells as early-stage apoptotic cells, aCasp3^+^ PI^+^ cells as late-stage apoptotic cells, and aCasp3^−^ PI^+^ cells as necrotic cells (Figure 1I). While the aCasp3^−^ PI^+^ population includes necrotic cells, it may also contain cells that died via other mechanisms.

Terminal deoxynucleotidyl transferase dUTP nick end labeling (TUNEL) is commonly used to detect cell death in developing retinas.^27,33^ We found that TUNEL marks both a fraction of apoptotic cells and necrotic cells in day 60 organoids, suggesting that TUNEL is not ideal for distinguishing between apoptosis and necrosis for this study (Figures S4A and S4B).

We first examined the regional distribution of apoptotic and necrotic cells. In day 60 organoids, 65% of apoptotic cells were localized to the retinal layers, while 88% of necrotic cells were found in the innermost core of the OVs (Figure S4C), suggesting that apoptosis primarily occurs in the retinal layers, while necrosis predominantly affects cells in the core. For subsequent analysis, we focused on apoptotic cells in the retinal layers and necrotic cells in the core.

We next examined the relative temporality of apoptosis and necrosis in retinal organoids. To track cell death in an agematched cell population, we pulsed phase 2 organoids with EdU (5-ethynyl-2′-deoxyuridine) for 24 h on day 26 and quantified apoptosis and necrosis in the EdU^+^ cells 4, 14, 24, and 44 days later (Figures S4D-S4F). Whereas apoptosis is highest 4 days after cell birth and decreases over time, necrosis peaks 14 days after cell birth and then decreases (Figure S4F). These data suggest that apoptosis occurs rapidly after cell birth, whereas death by necrosis is delayed.

To investigate the dynamics of cell death during organoid development, we quantified apoptotic and necrotic cells in OVs on days 32, 60, 80, 100, 130, and 200, spanning the three phases of RGC development and loss (Figures 1J-1O).

We first evaluated the overall densities of apoptotic cells (aCasp3^+^ PI^−^ and aCasp3^+^ PI^+^ cells) in the retinal layers during organoid development. Phase 1 organoids displayed the highest density of apoptotic cells (Figures 1J and 1K; day 32), suggesting an early wave of apoptosis. The density of apoptotic cells decreased in phase 2 (Figures 1J and 1K; days 60 and 80) and increased again in early phase 3 (Figures 1J and 1K; day 100), forming a late wave of apoptosis. The density decreased later in phase 3 (Figures 1J and 1K; days 130 and 200). These early and late waves of apoptosis in retinal layers of OVs (Figure 1K) resemble the two waves of developmental apoptosis in human (Figure S1) and other vertebrate retinas.^12-14,16-18^

We then assessed the apoptosis of RGCs during organoid development by quantifying aCasp3^+^ mNeon^+^ RGCs. Phase 1 organoids displayed a low proportion of apoptotic mNeon^+^ RGCs (Figures 1J′ and 1L; day 32). The proportion and density of apoptotic mNeon^+^ RGCs increased in phase 2 and early phase 3 (Figures 1J′, 1L, and S4G; days 60, 80, and 100), followed by their decrease later in phase 3 (Figures 1J′, 1L, and S4G; days 130 and 200). H7 *POU4F2-tdTomato* organoids displayed similar temporal patterns of apoptosis, showing reproducibility across cell lines (Figures S4H-S4J). The highest proportion of apoptotic mNeon^+^ RGCs coincides with the increase in overall apoptosis on day 100 (Figures 1K and 1L), suggesting that apoptosis of RGCs contributes to the late wave of apoptosis in organoids.

We next evaluated the overall densities of aCasp3^−^ PI^+^ necrotic cells in the core during organoid development. A small number of necrotic cells were observed in phase 1 (Figures 1M and 1N; day 32). The density of necrotic cells increased rapidly in phase 2 and early phase 3 (Figures 1M and 1N; days 60, 80, and 100), then decreased later in phase 3 (Figures 1M and 1N; days 130 and 200). These results demonstrate one wave of necrosis in the core of OVs during organoid development.

We then assessed the necrosis of RGCs by quantifying aCasp3^−^ PI^+^ mNeon^+^ RGCs. Few necrotic mNeon^+^ RGCs were observed in phase 1 (Figures 1M′ and 1O; day 32). The proportion and density of necrotic mNeon^+^ RGCs then increased in phases 2 and 3 (Figures 1M′, 1O, and S4K; days 60, 80, 100, and 130), followed by a sharp decrease in late phase 3 (Figure 1M′, 1O, and S4K; day 200). These results suggest that mNeon^+^ RGCs undergo significant necrosis between phases 2 and 3 in organoids.

To evaluate the contribution of apoptosis or necrosis to the loss of RGCs in retinal organoids, we compared the proportions of apoptotic and necrotic mNeon^+^ RGCs. Whereas <5% of mNeon^+^ RGCs were apoptotic (Figure 1L), 10%–59% of mNeon^+^ RGCs were necrotic between days 60 and 130 (Figure 1O). Moreover, this period of high-level RGC necrosis coincides with the decline of mNeon^+^ RGC density (Figure 1G), suggesting that necrosis is a predominant driver of RGC loss in human retinal organoids.

### Neurogenic RPCs and newborn neuronal precursors die during the early wave of apoptosis

Since RGC death occurs during the second wave of apoptosis and the wave of necrosis, other cell types likely die during the early wave of apoptosis. During the early wave of apoptosis in the chick retina, TUNEL^+^ cells were identified as proliferating neuroepithelial cells or migrating newborn RGC precursors.^15,19,53,78^ Considering the similar localization of apoptotic cells in the NBL of human fetal retinas (Figure S1A′) and organoids (Figure 1J), we hypothesized that these apoptotic cells are RPCs or postmitotic neuronal precursors.

To label the cell types contributing to the early wave of apoptosis, we differentiated retinal organoids from a human induced pluripotent stem cell (iPSC) line carrying the transgenic nuclear reporter *SIX6-p2A-H2B-GFP* (IMR90.4 *SIX6-H2B-GFP*).^79^ In organoid-derived OVs, H2B-GFP permanently labels all cells that expressed SIX6, including RPCs and their postmitotic neuronal progeny (Figure 1P).^80-82^

To test if the *SIX6-H2B-GFP* reporter marks the apoptotic cells during the early wave, we measured the densities of aCasp3^+^ GFP^+^ cells in the retinal layers (i.e., NBL and GCL) of IMR90.4 *SIX6-H2B-GFP* organoids on days 25, 30, 40, 60, and 80. We observed a peak of apoptotic aCasp3^+^ GFP^+^ cells on day 40 (Figure 1Q), validating our previous observation that the early wave of apoptosis peaked between days 30 and 40 (Figure 1K).

We next evaluated the distribution of apoptotic aCasp3^+^ GFP^+^ cells in the NBL and GCL during early organoid development. On days 25 and 30, nearly all aCasp3^+^ GFP^+^ cells were found in the NBL (Figures 1R and 1S). During the formation of the GCLs over 50% of aCasp3^+^ GFP^+^ cells were observed in the GCL on days 40, 60, and 80 (Figures 1R and 1S). These data suggest that the early wave of apoptosis comprises RPCs in the NBL first and immature neuronal precursors migrating to the developing GCL later.

To determine the contribution of RPCs to the early wave of apoptosis, we evaluated the proportion of aCasp3^+^ GFP^+^ cells expressing the RPC marker VSX2 on days 30 and 40. 6.8% of aCasp3^+^ GFP^+^ cells were VSX2^+^ on day 30, while the percentage dropped to 2.7% on day 40 (Figures S4L-S4N), suggesting that the contribution of RPCs in the early wave of apoptosis decreases as the GCL starts to develop. In day 30 organoids derived from a different stem cell line, we observed apoptotic cells that expressed the RPC marker PAX6 or VSX2 or the RGC marker ISL1/2 (Figures S4O-S4R). These results suggest that RPCs contribute to the early wave of apoptosis prior to the formation of the GCL.

At the beginning of retinal neurogenesis, the NBL consists of RPCs—including proliferative RPCs (pRPCs) that highly express cell-cycle-phase-associated genes and neurogenic RPCs (nRPCs) that upregulate proneural transcription factors for potential neurogenic division—as well as postmitotic neuronal precursors.^83-85^ To determine whether the apoptotic cells in the NBL are pRPCs, nRPCs, and/or neuronal precursors, we pulsed organoids with EdU for 24 h on day 26 and measured the proportion of apoptotic aCasp3^+^ GFP^+^ cells that were Ki67^+^ (pRPCs) or EdU^+^ (recently divided pRPCs, nRPCs, and newborn neuronal precursors) on day 30 (Figures S4S-S4U). 41.1% of aCasp3^+^ GFP^+^ cells were EdU^+^ (Figures S4S and S4T), while only 3.7% were Ki67^+^ (Figures S4S and S4U), suggesting that the early wave of apoptosis in human retinal organoids is composed primarily of nRPCs and/or newborn neuronal precursors, with a smaller contribution from pRPCs.

In summary, we observed an early wave of apoptosis during phase 1, a late wave of apoptosis in retinal layers during phase 2 and early phase 3, and a wave of necrosis in the core in phases 2 and 3 in human retinal organoids. These results suggest that the early wave of apoptosis mainly affects nRPCs and neuronal precursors, while the late wave of apoptosis and necrosis leads to the substantial loss of RGCs during long-term culture (Figure 1T).

### *BAX*/*BAK* knockout eliminates the two waves of apoptosis in retinal organoids

We next sought to investigate the roles of the two waves of apoptosis in human retinal organoids by inhibiting developmental apoptosis. BAX and BAK are pro-apoptotic proteins in the *BCL2* family that are essential regulators of the apoptotic pathway.^86^ In mouse retinas, genetically knocking out *Bax* and *Bak* inhibits developmental apoptosis, leading to increased thickness and cell numbers of the GCL and INL.^17,27^ CRISPR-edited genetic knockout of *BAX* and *BAK* in human iPSCs inhibits apoptosis (Figure S5A),^87^ providing an effective model to study the role of developmental apoptosis in human development.

To investigate how apoptosis affects retinal organoid development, we differentiated human retinal organoids from *BAX* and *BAK* double mutant (GM25256 *BAX*/*BAK dKO* [double knockout]) and isogenic control (GM25256) iPSCs (Figures S5B and S5C).^87^ *BAX*/*BAK dKO* and control organoids displayed no significant differences in the sizes of their OVs or their cores (Figures S5T and S5U). Organization of the retinal layers was similar throughout differentiation, except at day 200, when the retinal layers in *BAX*/*BAK dKO* organoids were significantly thicker (Figure S5V). These observations suggest that inhibition of apoptosis in *BAX*/*BAK dKO* organoids leads to improved long-term maintenance of retinal cells. Immunofluorescent staining confirmed the presence of major retinal neuronal types, indicating that *BAX*/*BAK dKO* does not block cell fate specification (Figure S5D-S5S).

Both control and *BAX*/*BAK dKO* organoids generated high-density outer layers (NBL and ONL) and sparser inner layers (intermingled INL+GCL). However, no inner plexiform layer formed to separate the INL and GCL during organoid long-term culture (Figure S5W). We observed a significant increase in the thickness of the INL+GCL in day 200 *BAX*/*BAK dKO* organoids, but not the ONL (Figures S5X-S5Z), suggesting that *BAX*/*BAK dKO* improves long-term survival of INL and GCL cells. Furthermore, ectopic rhodopsin^+^ cells were found in the inner retinal layers of day 200 *BAX*/*BAK dKO* organoids (Figure S5P and S5Q), similar to ectopic rods observed in the INL of *BAX*/*BAK dKO* mouse retinas.^27^ Together, these observations suggest that the changes in retinal layer structure in *BAX*/*BAK dKO* organoids are due to enhanced cell survival and altered migration.

**Figure 2.**
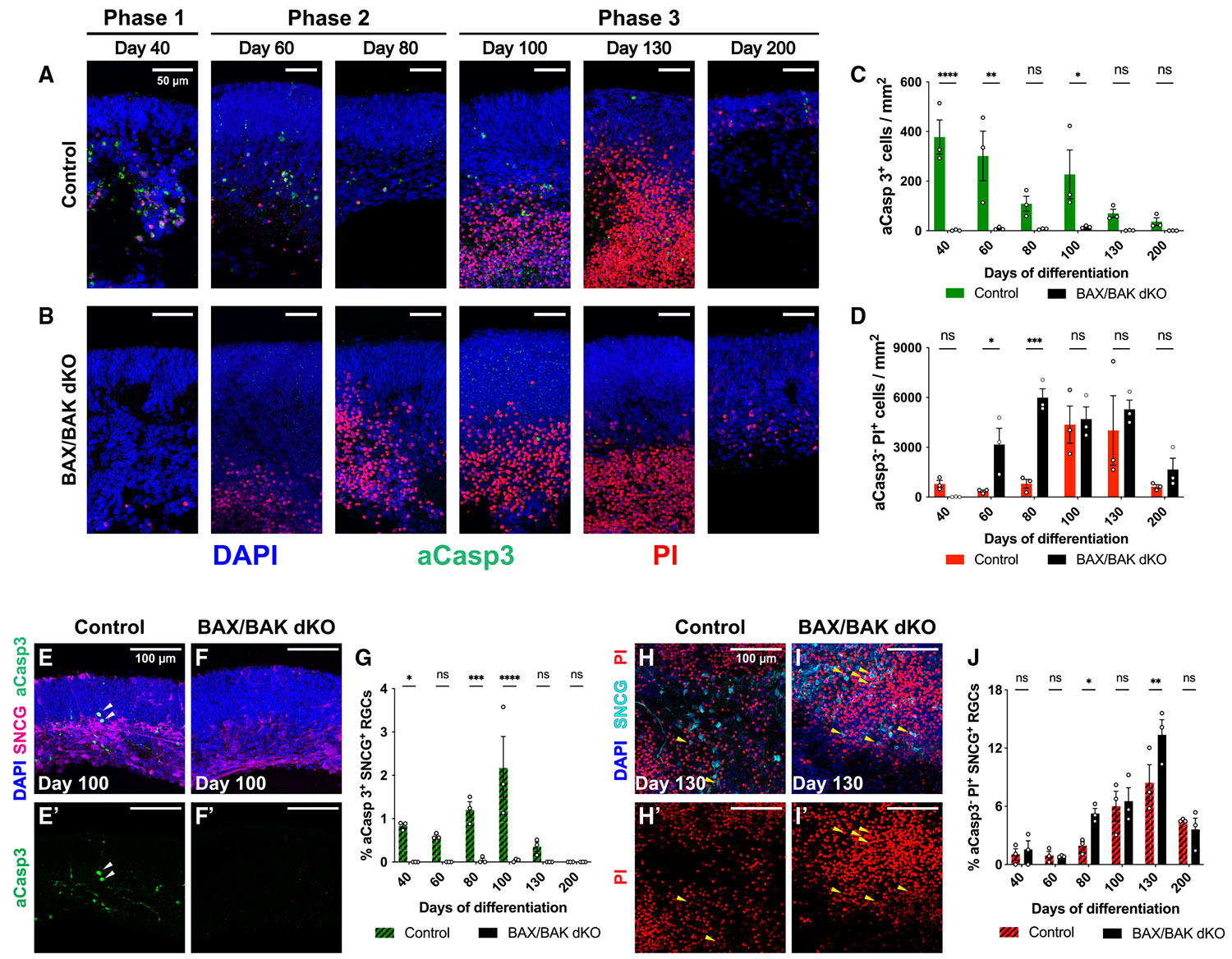
Apoptosis is blocked in *BAX*/*BAK* mutant organoids (A and B) aCasp3 (green) and PI (red) in control (A) and *BAX*/*BAK dKO* (B) organoids. Scale bar, 50 μm. (C and D) Density of aCasp3^+^ apoptotic cells in retinal layers (C) and aCasp3^+^ PI^−^ necrotic cells in the core (D) of control and *BAX*/*BAK dKO* organoids. Data are represented as the mean ± SEM. *n* = 3 organoids per time point. Two-way ANOVA followed by Sidá k’s post hoc test was used. *p < 0.05, **p < 0.01, ***p < 0.001, ****p < 0.0001, and ns, not significant. (E and F) aCasp3^+^ SNCG^+^ RGCs (white arrowheads) in control (E) and *BAX*/*BAK dKO* (F) retinal organoids. Scale bar, 100 μm. (G) Percentage of aCasp3^+^ SNCG^+^ RGCs of all SNCG^+^ RGCs in control and *BAX*/*BAK dKO* organoids. Data are represented as the mean ± SEM. *n* = 3 organoids per time point. Two-way ANOVA followed by Sidák’s post hoc test was used. *p < 0.05, ***p < 0.001, ****p < 0.0001, and ns, not significant. (H and I) PI^+^ SNCG^+^ RGCs (yellow arrowheads) in control (H) and *BAX*/*BAK dKO* (I) organoids. Scale bar, 100 μm. (J) Percentage of aCasp3^−^ PI^+^ SNCG^+^ RGCs of all SNCG^+^ RGCs in control and *BAX*/*BAK dKO* organoids. Data are represented as the mean ± SEM. *n* = 3 organoids per time point. Two-way ANOVA followed by Sidák’s post hoc test was used. *p < 0.05, **p < 0.01, and ns, not significant.

To assess how *BAX*/*BAK dKO* affects apoptosis in organoids, we quantified the densities of apoptotic cells (aCasp3^+^ PI^−^ and aCasp3^+^ PI^+^) (Figures 2A-2C). Control organoids displayed an early wave of apoptosis on days 40 and 60 and a late wave of apoptosis on day 100 (Figures 2A and 2C), similar to our observations in organoids derived from other cell lines (Figures 1J, 1K, and S4E), while *BAX*/*BAK dKO* organoids displayed low densities of apoptotic cells at all time points (Figures 2B and 2C). These results suggest that *BAX*/*BAK dKO* essentially abolishes the two waves of apoptosis in retinal organoids.

To determine how *BAX*/*BAK dKO* affects necrosis in organoids, we measured the density of necrotic cells (Casp3^−^ PI^+^) in the core during differentiation (Figures 2A, 2B, and 2D). In control organoids, the density of necrotic cells was low in phases 1 and 2 on days 40, 60, and 80, increased in phase 3 on days 100 and 130, and then decreased on day 200 (Figures 2A and 2D). In *BAX*/*BAK dKO* organoids, the density of necrotic cells was low at day 40 but increased during an extended wave of necrosis in phases 2 and 3 from day 60 to 130, followed by a decrease on day 200 (Figures 2B and 2D). Necrotic cell density was significantly higher in *BAX*/*BAK dKO* organoids during phase 2 on days 60 and 80 (Figure 2D), suggesting that loss of apoptosis leads to earlier and more extensive necrosis.

SNCG (γ-synuclein) is highly expressed in developing and mature mammalian RGCs.^88-90^ To validate if *BAX*/*BAK dKO* abolishes apoptosis of RGCs, we measured the proportion of apoptotic RGCs (aCasp3^+^ SNCG^+^) among all SNCG^+^ RGCs (Figures 2E-2G). In control organoids, apoptosis of RGCs peaked on day 100 in early phase 3 (Figures 2E and 2G), similar to our observations in organoids derived from other cell lines (Figures 1L and S4I). In contrast, apoptotic RGCs were nearly undetectable in *BAX*/*BAK dKO* organoids across all time points (Figures 2F and 2G). Thus, apoptosis of RGCs is blocked in *BAX*/*BAK dKO* organoids.

To determine how *BAX*/*BAK dKO* affects necrosis of RGCs, we measured the proportion of necrotic RGCs (aCasp3^−^ PI^+^ SNCG^+^) in the core among all SNCG^+^ RGCs (Figures 2H-2J). In control organoids, necrotic RGCs were rare in phases 1 and 2 (Figure 2J). Their proportion increased in early phase 3 on days 100 and 130 and decreased on day 200 (Figures 2H and 2J). In *BAX*/*BAK dKO* organoids, the proportion of necrotic RGCs was low on days 40 and 60 and increased later on days 80, 100, and 130, followed by a decline in late phase 3 on day 200 (Figures 2I and 2J). Necrosis of RGCs was elevated in *BAX*/*BAK dKO* organoids on days 80 and 130 (Figure 2J), suggesting that blocking apoptosis leads to increased necrosis of RGCs.

Taken together, inhibition of BAX/BAK-mediated apoptosis blocks the two waves of apoptosis, yields increased retinal layer thickness, and increases necrosis during long-term organoid culture.

### Blocking apoptosis increases nRPC and RGC abundance in retinal organoids

**Figure 3.**
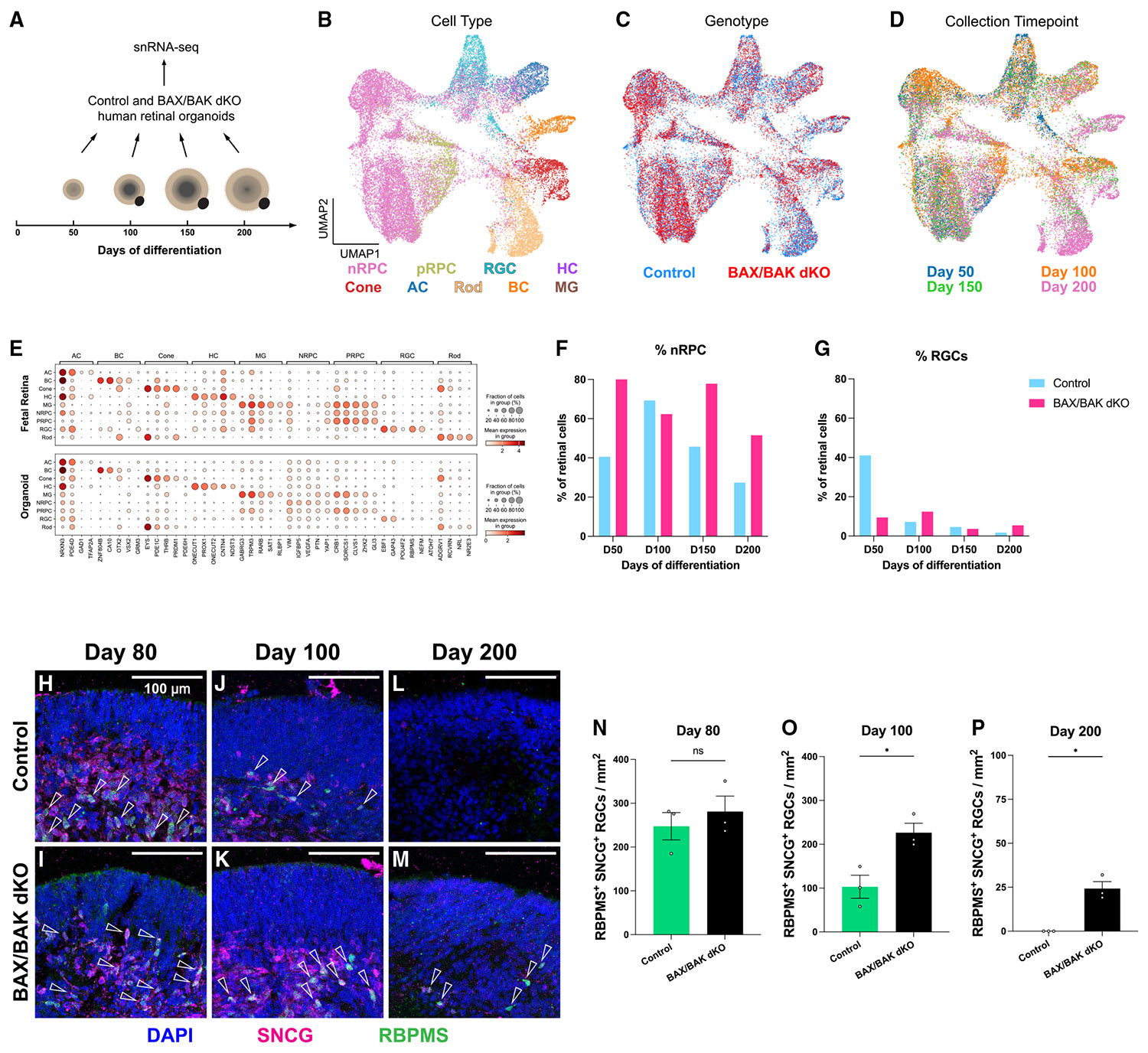
Blocking apoptosis leads to increases in nRPCs and RGCs in organoids (A) Design of snRNA-seq experiments. Control and *BAX*/*BAK dKO* retinal organoids were collected on days 50, 100, 150, and 200. (B–D) Uniform manifold approximation and projection (UMAP) of retinal organoid-derived snRNA-seq data, colored by retinal cell types (B), genotypes (C), or collection time points (D). nRPC, neurogenic RPC; pRPC, proliferative RPC; RGC, retinal ganglion cell; HC, horizontal cell; Cone, cone photoreceptor; AC, amacrine cell; Rod, rod photoreceptor; BC, bipolar cells; MG, Müller glia. (E) Validation of marker genes for retinal cell types in the fetal retina reference dataset (top) and retinal organoid dataset (bottom). (F and G) Percentage of annotated nRPCs (F) and RGCs (G) of retinal cells at each collection time point. Blue, control; red, *BAX*/*BAK dKO*. (H–P) Maintenance of RBPMS^+^ SNCG^+^ RGCs (empty arrowheads) in control (H, J, and L) and *BAX*/*BAK dKO* (I, K, and M) organoids on days 80, 100, and 200. Scale bar, 100 μm. (N–P) RBPMS^+^ SNCG^+^ RGC density in retinal layers of control and *BAX*/*BAK dKO* organoids on days 80 (N), 100 (O), 200 (P). Data are represented as the mean ± SEM. *n* = 3 organoids per time point. *p < 0.05 and ns, not significant; displayed *p* values were determined by an unpaired two-tailed Student’s *t* test (N and O) or an unpaired two-tailed Welch’s *t* test (P).

To assess how blocking apoptosis affects retinal organoid development, we performed 10× Chromium snRNA-seq on control and *BAX*/*BAK dKO* organoids collected on days 50, 100, 150, and 200 (Figure 3A). At each time point, four control and four *BAX*/*BAK dKO* organoids from the same batch were collected. After quality control and filtering, we profiled 29,821 nuclei in total. For dimensionality reduction, we applied non-negative matrix factorization (NMF) to identify gene expression patterns in our dataset (Figure S6A).^91,92^ Because human retinal organoids can also generate retinal pigmented epithelium (RPE) cells and other non-retinal cell types (e.g., brain and spinal cord-like cells),^93^ we manually annotated these populations using known markers and excluded them from downstream analysis (Figures S6B-S6D).

To annotate retinal cell types in our dataset, we trained a random forest classifier on a published single-nucleus dual-omics atlas of the human fetal retina as the reference (Figures S6E-S6H).^94,95^ Cell-type annotations for organoid-derived cells were then reprojected for visualization (Figure 3B). All major retinal cell types were present for control and *BAX*/*BAK dKO* organoids (Figures 3B-3D and S6I). Organoid-derived and fetal retinal cells exhibited highly similar expression of canonical marker genes and classifier-derived markers that define retinal cell types (Figure 3E),^95,96^ validating the cell-type annotations of the organoid dataset.

To determine how blocking apoptosis affects the abundance of retinal cell types, we compared the proportions of cell types. As the early wave of apoptosis affects nRPCs and newborn neuronal precursors (Figures 1S and S4S-S4U), we first compared the proportions of cells classified as nRPCs, which include dividing nRPCs and newborn precursors. *BAX*/*BAK dKO* organoids displayed higher proportions of nRPCs on days 50, 150, and 200 (Figure 3F). In contrast, differences in the proportions of pRPCs were minimal (Figures S6I-S6L). These results suggest that blocking apoptosis leads to an increase in nRPCs, consistent with our findings that the early wave of apoptosis affects nRPCs and newborn neuronal precursors (Figures S4S-S4U).

As the late wave of apoptosis affects RGCs, we next compared the proportions of RGCs. Surprisingly, *BAX*/*BAK dKO* organoids displayed a lower percentage of RGCs on day 50 (Figure 3G), suggesting that early RGC neurogenesis is delayed when apoptosis is blocked. Nevertheless, *BAX*/*BAK dKO* organoids exhibited a comparable or increased proportion of RGCs on days 100, 150, and 200 (Figure 3G), suggesting that RGC abundance is increased in *BAX*/*BAK dKO* organoids.

To validate these observations, we quantified RGC density in control and *BAX*/*BAK dKO* organoids on days 80, 100, and 200 of differentiation. RGCs were identified based on the co-expression of RGC markers SNCG and RBPMS. All SNCG^+^ RBPMS^+^ RGCs resided in the inner layers of the OV (Figures 3H-3M). SNCG^+^ RBMPS^+^ RGC density was similar in control and *BAX*/*BAK dKO* organoids in phase 2 on day 80 (Figures 3H, 3I, and 3N). In contrast, the densities of SNCG^+^ RBMPS^+^ RGCs were significantly increased in *BAX*/*BAK dKO* organoids in phase 3 on days 100 and 200 (Figures 3J-3M and 3O-3P). Similarly, *BAX*/*BAK dKO* organoids displayed an increased density of POU4F1^+^ RGCs on day 200 (Figures S6M-S6O). These data show that blocking apoptosis increases RGC abundance during long-term organoid culture.

To investigate how blocking apoptosis affected the integration of RGCs into the retinal layers, we stained for SNCG^+^ RGCs and CHAT^+^ SACs, which reside in the inner retinal layers of organoids. Both markers label the soma and neurites, allowing visualization of potential cell-cell contacts between RGCs and SACs. On day 100, both RGCs and SACs extended their neurites in proximity in control and *BAX*/*BAK dKO* organoids (Figures S6P and S6S), indicating potential cell-cell interactions. In control organoids, SACs displayed fewer neurites on day 200 following the loss of RGCs in late phase 3 (Figure S6Q and S6R). In *BAX*/*BAK dKO* organoids, more surviving SNCG^+^ RGCs remained in the vicinity of SACs on days 130 and 200, yet RGCs and SACs showed few neurites (Figure S6T and S6U), suggesting that these surviving RGCs are unlikely to form additional synaptic interactions with other retinal neurons in *BAX*/*BAK dKO* organoids.

Together, blocking apoptosis increases the abundance of nRPCs/neuronal precursors and RGCs, consistent with their death in the early and late waves of apoptosis, respectively, but is unlikely to yield interactions of RGCs with other retinal neurons in long-term organoid culture.

### Blocking apoptosis delays early RGC neurogenesis and improves RGC survival in retinal organoids

Since apoptosis affects nRPCs/neuronal precursors and RGCs, the increased density of RGCs in phase 3 *BAX*/*BAK dKO* organoids may result from increased RGC neurogenesis and/or improved RGC survival.

**Figure 4.**
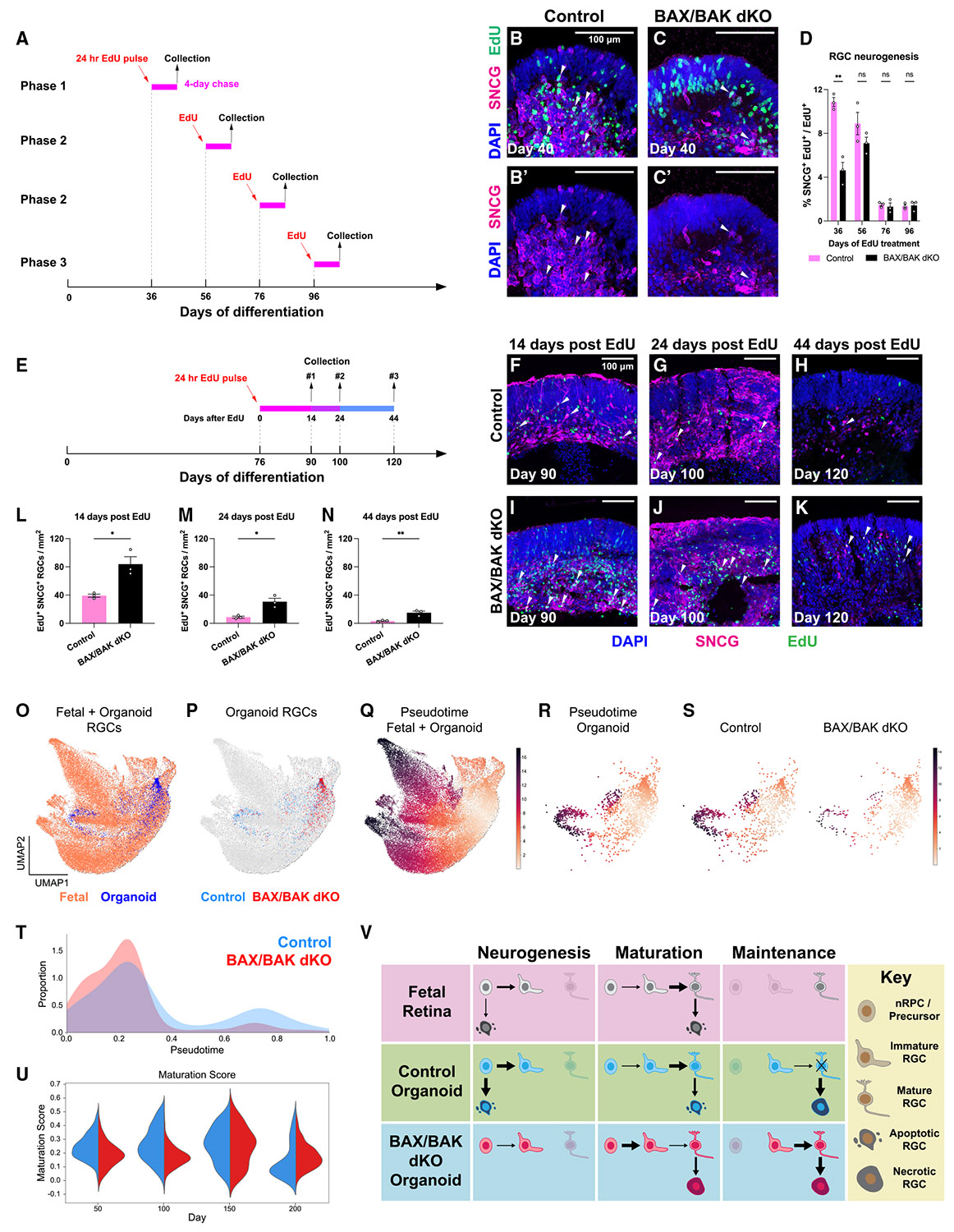
Blocking apoptosis delays RGC development and promotes RGC survival in organoids (A–D) *BAX*/*BAK dKO* delays RGC neurogenesis. (A) Experimental design of RGC neurogenesis measurement by EdU labeling. (B and C) EdU^+^ SNCG^+^ RGCs (white arrowheads) in day 40 control (B) and *BAX*/*BAK dKO* (C) organoids. Scale bar, 100 μm. (D) Percentage of EdU^+^ SNCG^+^ RGC of all EdU^+^ cells in control and *BAX*/*BAK dKO* organoids following EdU treatment on days 36, 56, 76, and 96. Data are represented as the mean ± SEM. *n* = 3 organoids per time point. An unpaired two-tailed Student’s *t* test was used at each time point; **p < 0.01 and ns, not significant. (E–N) *BAX*/*BAK dKO* promotes RGC survival. (E) Experimental design of RGC lifespan measurement by EdU labeling. (F–K) EdU^+^ SNCG^+^ RGCs (white arrowheads) in control (F–H) and *BAX*/*BAK dKO* (I–K) organoids 14, 24, and 44 days after EdU treatment. Scale bar, 100 μm. (L–N) Density of EdU^+^ SNCG^+^ RGCs 14 (L), 24 (M), and 44 (N) days after EdU treatment. Data are represented as the mean ± SEM. *n* = 3 organoids per time point. An unpaired two-tailed Student’s *t* test was used at each time point; *p < 0.05, **p < 0.01, and ns, not significant. (O and P) Integrated UMAP maps of fetal and organoid-derived RGC snRNA-seq data colored by sample sources (O; orange, fetal retina; blue, organoid) or genotypes (P; blue, control organoid RGCs; red, *BAX*/*BAK dKO* organoid RGCs; gray, fetal retina RGCs). (Q) Integrated UMAP of fetal and organoid RGCs is colored by pseudotime. (R and S) UMAPs of all organoid RGCs (R), control organoid RGCs (S, left), and *BAX*/*BAK dKO* organoid RGCs (S, right) colored by pseudotime. (T) Proportional distribution of control (blue) and *BAX*/*BAK dKO* (red) organoid RGCs along the inferred pseudotime trajectory. (U) Violin plots of maturation scores of control (blue) and *BAX*/*BAK dKO* (red) organoid RGCs on days 50, 100, 150, and 200. (V) Model for cell death and RGC development in human fetal retinas and retinal organoids.

To test how blocking apoptosis affects RGC neurogenesis, we pulsed control and *BAX*/*BAK dKO* organoids with EdU for 24 h on days 36, 56, 76, and 96 and measured the percentage of newborn EdU^+^ SNCG^+^ RGCs among all EdU^+^ cells 4 days later (Figures 4A-4D). The proportion of EdU^+^ SNCG^+^ RGCs was higher in control compared to *BAX*/*BAK dKO* organoids following pulses on day 36 (Figures 4B-4D) but not on day 56 (Figure 4D), suggesting that blocking apoptosis delays early RGC neurogenesis. In contrast, both genotypes showed similarly low proportions when pulsed on days 76 and 96 (Figure 4D), indicating that blocking apoptosis does not extend the time window of RGC neurogenesis in organoids. We validated this temporal pattern of RGC neurogenesis with a separate stem cell line (Figures S7A-S7C). In addition, we found no evidence of cytotoxic effects of day 76 EdU treatment in phase 2 organoids (Figures S7D and S7E), indicating that the decreased EdU^+^ RGC proportion is not caused by EdU-induced cell death.^97^

To test whether blocking apoptosis improves RGC survival, we pulsed control and *BAX*/*BAK dKO* organoids with EdU for 24 h on day 76 and measured the densities of EdU^+^ SNCG^+^ RGCs 14, 24, or 44 days later (Figure 4E). Control organoids displayed a near-complete loss of EdU^+^ SNCG^+^ RGCs by day 44 after the EdU pulse, suggesting that most RGCs born on day 76 have a lifespan shorter than 44 days (Figures 4F-4H). We validated the lifespan of RGCs with a separate stem cell line (Figures S7F-S7J). In contrast, *BAX*/*BAK dKO* organoids displayed higher densities of EdU^+^ SNCG^+^ RGCs compared to the control organoids on days 14, 24, and 44 after the EdU pulse (Figures 4I-4K and 4L-4N), suggesting that RGC survival is improved in *BAX*/*BAK dKO* organoids.

Taken together, blocking apoptosis delays early RGC neurogenesis and improves RGC survival in *BAX*/*BAK dKO* retinal organoids.

### Blocking apoptosis slows maturation but promotes survival of mature RGCs

We next investigated RGC maturation to understand how apoptosis regulates overall RGC development. To compare RGC development in human retinal organoids and fetal retinas, we used transfer learning to project annotated organoid-derived RGCs onto the uniform manifold approximation and projection (UMAP) of human fetal RGCs from the reference snRNA-seq dataset (Figures 4O-4P).^91,95^

To evaluate the progression of RGC maturation, we performed pseudotime analysis on fetal- and organoid-derived RGCs using scFates (Figure 4Q).^98^ The inferred pseudotime trajectory highly aligns with the progression of biological ages in human fetal RGCs (Figures S7K-S7M), suggesting that the pseudotime trajectory approximates *in vivo* human RGC maturation. Human organoid-derived RGCs show similar pseudotime trajectories (Figure 4R), validated by the expression of genes associated with different maturation stages of RGCs (*ATOH7*, *GFRA1*, and *FGF12*) (Figure S7N).^99-103^

To assess how blocking apoptosis affects the progression of RGC maturation, we compared the distribution of RGCs from *BAX*/*BAK dKO* and control organoids along the pseudotime trajectory (Figure 4S). Control organoids showed a greater proportion of RGCs at later pseudotime, whereas *BAX*/*BAK dKO* organoids were enriched for RGCs at early pseudotime (Figure 4T). These results suggest that blocking apoptosis impedes the maturation of RGCs.

To determine how the transcriptome changes during human RGC maturation, we used scFates to identify a set of 142 genes associated with mid-late and late stages of RGC maturation from a human fetal retina dataset (Figure S7O; Table S1). Expression of these genes generally correlated with pseudotime progression of RGCs in control and *BAX*/*BAK dKO* organoids (Figure S7P and S7Q). To quantify the expression of these maturation-associated genes, we then calculated an aggregate gene expression score (“maturation score”) for the same gene set.^104^ The maturation score increased with the biological ages of fetal-derived RGCs (Figure S7R), suggesting that it captures the developmental progression of human fetal RGCs.

To assess how blocking apoptosis affects transcriptomic changes during RGC maturation, we compared maturation scores of RGCs from control and *BAX*/*BAK dKO* organoids on days 50, 100, 150, and 200. On days 50 and 100, *BAX*/*BAK dKO* RGCs showed lower maturation scores than control RGCs (Figure 4U), consistent with delayed RGC neurogenesis and maturation upon inhibition of apoptosis (Figures 4B-4D and 4T). On day 150, maturation scores were comparable between the two genotypes (Figure 4U). On day 200, *BAX*/*BAK dKO* RGCs exhibited higher maturation scores than control RGCs (Figure 4U), suggesting that blocking apoptosis leads to improved survival of more mature RGCs, consistent with our immunofluorescent staining results (Figures 3H-3P).

Taken together, our data suggest that RGCs in control organoids are generated, mature, and die by 200 days of differentiation, while RGCs in *BAX*/*BAK dKO* organoids are generated and mature more slowly but survive more readily until day 200.

## DISCUSSION

We characterize here the spatiotemporal dynamics of cell death in human fetal retinas and retinal organoids, develop a strategy to promote RGC long-term survival in organoids by blocking apoptosis, and reveal a regulatory role of developmental apoptosis in RGC development. Human fetal retinas exhibit two waves of developmental apoptosis, with the first wave affecting nRPCs and neuronal precursors and the second wave targeting RGCs and other retinal neurons (Figure 4V). Human retinal organoids similarly display two waves of apoptosis, as well as an additional wave of necrosis (Figure 4V). When apoptosis is blocked in organoids, RGC neurogenesis and maturation are delayed, while their survival is improved (Figure 4V). These findings provide new insights into the roles of apoptosis in human RGC development and unveil promising strategies to promote RGC long-term survival in organoid models for future therapeutic applications.

### Comparing developmental cell death between human retinas and retinal organoids

Our studies reveal key similarities and differences in developmental apoptosis between human retinas and organoids. The patterns of apoptosis are broadly similar. In both fetal retinas and retinal organoids, an early wave of apoptosis affects RPCs and neural precursors, followed by a late wave that selectively eliminates a subset of RGCs and other neurons. At the peaks of the late wave, ~1.0% of RBPMS^+^ RGCs were apoptotic in human fetal retinas (Figure S1H), whereas ~4.4% of mNeon^+^ RGCs were apoptotic in organoids (Figure 1L). Though apoptosis plays a central part in regulating RGC development in both contexts, necrosis emerges as an additional mechanism of cell death, causing RGC loss during long-term organoid culture (Figure 1O).

These results suggest that organoids may lack essential extrinsic factors required for sustained RGC survival and maturation, such as neurotrophic factors,^105^ physiological electrical stimuli,^106-108^ and proper oxygen supply.^109,110^ Further investigation will focus on recapitulating the supporting factors present in the human fetal environment during organoid culture.

In addition, *in vivo* retinas have mechanisms that kill cells and clear dead cells that are absent in organoids. Immune cells, especially microglia, play an important role in developmental cell death in the retina.^111,112^ In embryonic mouse retinas, microglia regulate RGC density by phagocytosing a subset of newborn, non-apoptotic RGCs but do not affect RGC neurogenesis or the early apoptotic wave.^113^ During the late apoptotic wave in postnatal mouse retinas, microglia clear apoptotic RGCs depending on the recognition receptors CR3 and Mer.^114^ Microglia and other immune cells are absent in human retinal organoids generated by the current protocols.^115^ Thus, cell-cell interactions and clearance of dead cells during the apoptotic waves in organoids occur in the absence of these *in vivo* mechanisms.

### Developmental apoptosis regulates RGC neurogenesis and maturation

Blocking the early wave of apoptosis in nRPCs and neural precursors leads to an increased population of nRPCs and delays in RGC neurogenesis and maturation, suggesting a non-cell-autonomous mechanism regulates RGC production. Our findings suggest that the early wave of apoptosis lowers the RPC-to-neuron ratio by eliminating nRPCs and neuronal precursors, promoting RGC neurogenesis and retinal development. Blocking apoptosis shifts the ratio toward nRPCs/neuronal precursors by increasing their relative abundance to neurons, which delays RGC neurogenesis and maturation. In the developing mouse retina, inhibition of apoptosis by *Bax* and *Bak* knockout does not increase cell proliferation.^27,28^ Mouse RPC proliferation and differentiation are regulated by RGC numbers via the Shh signaling pathway, highlighting a critical role of the RPC-to-neuron ratio in coordinating the timing of retinal development.^116,117^ Recently, we found a similar role for the RPC-to-neuron ratio in regulating photoreceptor developmental timing via thyroid hormone levels and the dynamic expression in RPCs of DIO3, a thyroid-hormone-degrading enzyme.^118^ It appears that the shifting RPC-to-neuron ratio plays an active role in regulating the timing of retinal neurogenesis and other developmental mechanisms.

Blocking the late wave of RGC apoptosis leads to an increased RGC population in human retinal organoids. In developing mouse retinas, blocking developmental apoptosis by global or RGC-specific *Bax* knockout increases RGC density.^17,119-121^ Notably, *Bax* knockout not only elevates the number of melanopsin-expressing intrinsically photosensitive RGCs (ipRGCs) but also alters their clustering and dendritic fasciculation.^122,123^ Although Bax-deficient mice exhibit normal RGC axonal integrity, non-image-forming target projection, and circadian photoentrainment compared to the wild type, retinal circuitry to ipRGCs is disrupted.^123,124^ In *Bax* and *Bak* DKO mice, additional GCL cells are maintained, but the effects on RGC neurogenesis, maturation, and morphology were not evaluated.^27^

Our findings in human retinal organoids recapitulate many aspects of apoptosis-mediated regulation observed in mice. Future studies should compare our observations in human retinal organoids with mouse models to determine whether any observed differences are species-specific or due to differences between *in vivo* and *in vitro* conditions.

### Promoting RGC survival in human retinal organoids

Different strategies have been applied to promote RGC survival in long-term organoid culture. Generation of assembloids by fusing human retinal organoids and brain organoids allows robust RGC neurite outgrowth, reduces apoptosis, and enhances the maintenance of RGCs through day 150. Similarly, providing brain-derived neurotrophic factor (BDNF) promotes RGC maintenance in organoids.^45^ Generation of vascularized human retinal organoids by co-culturing growing retinal organoids and epithelial cells reduces apoptosis and promotes the upregulation of RGC markers.^48^ In our study, genetic knockout of *BAX* and *BAK* blocks RGC apoptosis and prolongs the survival of SNCG^+^ RBPMS^+^ RGCs through day 200 of differentiation (Figures 3H-3P), highlighting the contribution of apoptosis to the progressive loss of RGC in organoids.

Although human retinal organoids cultured in bioreactors or on microfluidic chips display increased size, reduced hypoxic stress, and increased yields of photoreceptors and RGCs,^46,47,125,126^ how these strategies alter necrosis and long-term RGC survival remains unclear. Our study characterizes the formation of the necrotic core and how necrosis causes RGC loss (Figures 1M-1O, S3H, and S4K), underscoring the need to reduce necrosis in long-term organoid culture to improve the yield of mature RGCs. Air-liquid interface culture of brain organoid slices alleviates interior hypoxia and facilitates their expansion and lamination, making it a promising assay for reducing necrosis in the core of organoids.^71,127,128^ Future efforts will develop strategies combining inhibition of apoptosis with reduction of necrosis to further enhance RGC survival in retinal organoids, thereby advancing their translational potential for transplantation-based therapies of optic neuropathies.^129^

Taken together, this study deepens our understanding of the mechanisms that regulate human RGC neurogenesis, maturation, and survival. Moving forward, advances in organoid engineering that minimize loss and promote maturation of RGCs will be crucial for improving disease modeling and developing regenerative cell therapies for neurodegenerative diseases of the human visual system.

### Limitations of the study

The imaging-based co-staining assays conducted in this study cannot rule out the possibility that aCasp3^+^ PI^+^ apoptotic RGCs at late stages become aCasp3^−^ PI^+^ necrotic RGCs due to secondary necrosis and/or delayed clearance in retinal organoids. Real-time longitudinal tracking of dying RGCs across the full degeneration-clearance process will be essential to resolve the underlying mechanisms of RGC death and clearance in human retinal organoids. In addition, although we show that two waves of apoptosis occur in developing human retinas and retinal organoids, we cannot assess the contributions of immune cells (e.g., microglia) or retinal vasculature to developmental apoptosis, as these cells are absent from retinal organoids. Future studies incorporating these cells into organoid models may help address this limitation. Finally, we evaluate the maturation of RGCs in organoids by morphological characterization and single-nucleus transcriptomic analysis. Electrophysiological recordings from individual RGCs will enable the assessment of the functional maturity of surviving RGCs over long-term organoid culture.

## STAR★METHODS

### EXPERIMENTAL MODEL AND STUDY PARTICIPANT DETAILS

#### Human fetal samples

All human fetal samples were collected under Institutional Review Board Approval (STUDY-22-00065) at the Icahn School of Medicine at Mount Sinai. Consent for tissue donation was obtained after the patient had already made the decision for pregnancy termination and was obtained by a different clinical research coordinator than the physician performing the procedure. All tissues were deidentified, and the only clinical information collected was gestational age and the presence of any maternal or fetal diagnoses. All tissues were shipped in cold fixative. All fetal ages were converted to post-conception weeks in this study.

#### Human pluripotent stem cells

H7 *POU4F2-P2A-tdTomato-P2A-mTHY1.2* (modified H7 (WA07) human embryonic stem cells (hESCs), gift from the Zack lab at JHMI), H9 *POU4F2-P2A-mNeon-H2B* (modified H9 (WA09) hESCs, gift from the Wahlin lab at UCSD), IMR90.4 *SIX6-P2A-H2B-GFP* (modified hiPSCs, gift from the Zack lab at JHMI), wildtype GM25256 and *BAX/BAK dKO* GM25256 cells (modified hiPSCs, gift from the Gama lab at Vanderbilt University) were used for differentiation. Stem cells were maintained in mTeSR1 (Cat# 85857, StemCell Technologies) on 1% (v/v) Matrigel-GFR (Cat# 354230, BD Biosciences) coated dishes and grown at 37°C and with 10% CO_2_ + 5% O_2_ in a HERAcell 150i or 160i incubator (Thermo Fisher Scientific). Cells were passaged every 4–5 days according to confluence as in previous studies.^59^ Cells were passaged with Accutase (Cat# SCR005, Sigma) for 8–12 min and dissociated to single cells. Cells in Accutase were added 1:2 to mTeSR1 with 5 μM Blebbistatin (Bleb, Cat# B0560, Sigma), and pelleted at 150X gravity for 5 min. Cells were resuspended in mTeSR1 with 5 μM Blebbistatin, density was quantified, and they were plated at 5,000–15,000 cells per well in a prepared 6-well Matrigel-coated plate. Cells were fed with mTeSR1 48 h after passaging and every subsequent 24 h until passaged again. To minimize cell stress, no antibiotics were used. Cell lines were tested monthly for mycoplasma using MycoAlert (Cat# LT07, Lonza).

#### Cell culture media

##### E6 supplement:

970 μg/mL Insulin (Cat# 11376497001, Roche), 535 μg/mL holo-transferrin (Cat# T0665, Sigma), 3.20 mg/mL L-ascorbic acid (Cat# A8960, Sigma), 0.7 μg/mL sodium selenite (Cat# S5261, Sigma).

##### BE6.2 media:

2.5% E6 supplement (above), 2% B27 supplement (50X) minus Vitamin A (Cat# 12587010, Thermo Fisher Scientific), 1% Glutamax (Cat# 35050061, Thermo Fisher Scientific), 1% NEAA (Cat# 11140050, Thermo Fisher Scientific), 1 mM Sodium Pyruvate (Cat# 11360070, Thermo Fisher Scientific), and 0.87 mg/mL NaCl in DMEM (Cat# 11885084, Thermo Fisher Scientific).

##### LTR (Long-term retina) media:

25% F12 (Cat# 11765062, Thermo Fisher Scientific), 2% B27 supplement (50X) (Cat# 17504044, Thermo Fisher Scientific), 10% heat-inactivated FBS (Cat# 16140071, Thermo Fisher Scientific), 1mM Sodium Pyruvate (Cat# 11360070, Thermo Fisher Scientific), 1% NEAA (Cat# 11140050, Thermo Fisher Scientific), 1% Glutamax (Cat# 35050061, Thermo Fisher Scientific), and 1 mM taurine (Cat# T-8691, Sigma) in DMEM (Cat# 11885084, Thermo Fisher Scientific).

### METHOD DETAILS

#### Differentiation of human retinal organoids

Retinal organoids were differentiated from H7 ESCs as described^59^ with minor modifications (Figure S2). Briefly, human pluripotent stem cells were well-maintained, and only cultures with minimal to no spontaneous differentiation were used for aggregation. To aggregate, cells were passaged in Accutase at 37°C for 12 min to ensure complete single-cell dissociation. Cells were seeded in 50 μL of mTeSR1 at 3,000 cells/well into 96-well ultra-low adhesion round-bottom Lipidure coated plates (Cat# 51011610, NOF). Cells were placed in hypoxic conditions (10% CO_2_ and 5% O_2_) for 24 h to enhance survival. Cells naturally aggregated by gravity over 24 h.

On day 1, cells were moved to normoxic conditions (5% CO_2_). On days 1–3, 50 μL of BE6.2 media containing 3 μM Wnt inhibitor (IWR1e; Cat# 681669, EMD Millipore) and 1% (v/v) Matrigel were added to each well. Between days 4–9, 100 μL of media were removed from each well, and 100 μL of media were replenished. On days 4–5, 100 μL of BE6.2 media containing 3 μM Wnt inhibitor and 1% Matrigel was added. On days 6–7, 100 μL of BE6.2 media containing 1% Matrigel was added. On days 8–9, 100 μL of BE6.2 media containing 1% Matrigel and 100 nM Smoothened agonist (SAG; Cat# 566660, EMD Millipore) was added.

On day 10, cell aggregates (organoids) were transferred to 15 mL tubes, rinsed 2–3X in DMEM (Cat# 11885084, Thermo Fisher Scientific), and resuspended in BE6.2 media with 100 nM SAG in untreated 10 cm polystyrene petri dishes. From this point on, media was changed every other day. Organoids were monitored daily and manually separated if stuck together or to the plate.

On days 13–18, LTR media with 100 nM SAG was added.

On days 16–20, organoids were maintained in LTR, and washed 2X with 5 mL of DMEM before being transferred to new plates to wash off dead cells. To increase the yield of retinal tissue, 1 μM all-trans retinoic acid (ATRA; Cat# R2625, Sigma) was added to LTR medium from days 20–130 in all conditions. 10 μM Gamma-secretase inhibitor (DAPT; Cat# 565770, EMD Millipore) was added to LTR from days 28–42. Organoids were grown at low density (10–20 per 10 cm dish) to reduce aggregation.

Between days 20 and 40, optic vesicles-like structures (OVs) were manually dissected if needed using sharpened tungsten needles and collected. After dissection, organoids were transferred into 15 mL tubes and washed 2X with 3 mL of DMEM. After day 40, organoids were regularly culled if they failed to differentiate or maintain retinal tissue properly.

#### Phase quantification in retinal organoids

Human retinal organoids between days 30 and 200 of differentiation were kept in petri dishes with organoid culture media and live imaged with the EVOS XL Core Cell imaging system before collection. Differential interference contrast (DIC) and fluorescent images (555 nm channel) of developing organoids were overlayed by the default function of the EVOS XL Core imaging system and used for manual determination of RGC developmental phases based on RGC abundance and distribution. For quantification of RGC developmental phase progression, individual organoids were transferred and kept growing in designated wells of a 24-well plate separately between days 40 and 130 of differentiation. DIC and fluorescent images of these organoids were taken every 10 days, and their RGC developmental phases were evaluated as mentioned above.

#### Mycoplasma monitoring

Cell lines and organoid media were tested monthly for mycoplasma using MycoAlert (Cat# LT07, Lonza) and excluded if positive.

#### Cryosection

Fetal retinas and organoids were fixed by 4% formaldehyde for 1 h at room temperature (20°C–25°C), treated with a serial wash of 5% (3X quick rinse), 6.25% (for 30 min), 12.5% sucrose (for 30 min) in 0.1M PBS, and then left in 25% sucrose in 0.1M PBS at 4°C overnight. On the next day, human fetal retinas and organoids were flash frozen in OCT on dry ice and moved to −80°C for long-term storage. On the day of cryosectioning, samples were retrieved from −80°C to −20°C freezers to adapt to the new temperature for at least 30 min. Cryosectioning was performed on CryoStar NX50 (epredia). Fetal retinas were oriented and cryosectioned along the nasal-temporal axis at 12–16 μm. Organoids were cryosectioned at 10–12 μm. Sections were left to air-dry at room temperature for at least 3 h, and then stored long-term at −80°C.

#### Immunohistochemistry

Sections of human fetal retinas and organoids were retrieved from −80°C freezers, adapted to room temperature for 10 min, and then heated in HybEZ II Oven (ACDBio) at 50°C for 10-15 min to minimize remaining water droplets. Sections were washed quickly in 1X PBS to remove remaining OCT, then blocked and permeabilized in 10% donkey serum +0.5% Triton X-100 in PBS (blocking solution) for 1 h at room temperature. Sections were then incubated with primary antibodies (see key resources table) in blocking solution for 16–20 h or overnight at 4°C. Sections were washed 3X for 15 min in PBS, and incubated with secondary antibodies (Thermo Fisher Scientific, donkey secondary antibodies conjugated to Alexa Fluor 488, 555 or 647) diluted 1:800 in blocking solution for 1 h (2 h for fetal retina sections) at room temperature. Sections were then washed 3X in PBS quickly to get ready for nuclei counterstaining. For organoid samples, sections were incubated in 6 μM DAPI (4′,6-diamidino-2-phenylindole) in 1X PBS for 4 min at room temperature. For fetal retina samples, sections were incubated in 1:2000 dilution of Hoechst (Cat# 40046, Biotium) in PBS for 10 min at room temperature. Sections were then washed 3X for 15 min in PBS and mounted for imaging in SlowFade Gold Antifade mountant (Cat# P36940, Thermo Fisher Scientific).

#### Apoptosis and necrosis co-detection

Human retinal organoids were collected and put in 3.5 cm petri dishes (Cat# 229638, CELLTREAT) with organoid culture media. Propidium iodide (Cat# P4170, Sigma-Aldrich) was filter sterilized and diluted in culture media to the final concentration of 25 μg/mL. Organoids were incubated in propidium iodide-added organoid culture media for 12 h or overnight and collected on the next day. Treated organoids were then fixed, frozen, and cryosectioned for immunohistochemistry as mentioned above.

To distinguish apoptotic and necrotic cells in organoid sections, an antibody against cleaved/active Caspase 3 (Cat# 9961, Cell Signaling) was used for immunofluorescent staining. For quantitative analysis, aCasp3^+^ PI^−^ cells were counted as early apoptotic cells, and aCasp3^+^ PI^+^ cells were counted as late apoptotic cells, while aCasp3^−^ PI^+^ cells were counted as necrotic cells. Only apoptotic cells located in the retinal layers and necrotic cells located in the core were quantified in longitudinal analyses.

#### TUNEL assay

The Click-iT Plus TUNEL Assay (Cat# C10617, Invitrogen) was conducted on human retinal organoid sections based on the manufacturer’s instructions for tissue sections. Before the TUNEL assay, the sections were washed with 1X PBS for 3 times to remove remaining OCT. After the TUNEL assay, sections were handled following the immunohistochemistry protocol for primary and secondary antibody staining, starting from blocking and permeabilization as mentioned above, but protected from light to minimize photobleaching.

#### EdU treatment and detection

EdU solution (Click-iT EdU Assay, Cat# C10640, Invitrogen) was diluted in DMSO and added to the organoid culture media to a working concentration of 10 μM for 24 h in the incubator, before treated organoids were washed and transferred to EdU-free organoid culture media. Optimal concentration of EdU was adjusted based on the viability of cell lines and kept consistent within the same set of experiments.

The Click-iT Plus EdU Assay (Cat# C10640, Invitrogen) was conducted on human retinal organoid sections based on the manufacturer’s instructions for tissue sections. Before the EdU assay, sections were washed with 1X PBS for 3 times to remove remaining OCT. After the assay, sections were stained by primary and secondary antibodies following the immunohistochemistry protocol, starting from blocking and permeabilization as mentioned above, but with caution to minimize exposure to light and avoid photobleaching.

#### Microscopy and image processing

Brightfield images were acquired with an EVOS XL Core Cell imaging system. Differential Interference Contrast images were acquired with an EVOS M500 Imaging system on DIC with a 4x or a 10× air objective lens (Thermo Fisher). Fluorescent images were acquired using a Zeiss LSM 980 laser scanning confocal fluorescent microscopy. Confocal images were acquired with similar settings for laser power, photomultiplier gain and offset, and pinhole diameter. Camera images were acquired at similar epifluorescence intensity and exposure. Sections were imaged at 8–22 optical sections and 1–2 μm step size. Fluorescent images with a maximum intensity projection were used for downstream analysis.

#### Sequencing sample collection and nuclei lysis

4 control and 4 *BAX/BAK dKO* organoids with OVs were collected at each collection timepoint (days 50, 100, 150, and 200). Media was aspirated from the organoids and the tissue was submerged in liquid nitrogen to flash freeze. Organoid samples were stored at −80°C until library preparation, to reduce batch effect across samples. Nuclei were isolated from retinal organoids using the chilled lysis buffer (10 mM Tris-HCl, 10 mM NaCl, 3 mM MgCl_2_, 0.01% Tween 20, 0.01% Nonidet P-40, 0.001% Digitonin, 1mM DTT, 1% BSA). The homogenate was incubated on ice for 10 min, washed twice with a resuspension buffer (10 mM Tris-HCl, 10 mM NaCl, 3 mM MgCl_2_, 0.1% Tween 20, 1mM DTT, 1% BSA) and filtered through a series of 70 μm and 40 μm Flowmi filters. The nuclei were resuspended in an appropriate volume of diluted nuclei buffer (10X Genomics) to achieve a concentration of approximately 2000–5000 nuclei/μL. Nuclei concentration was determined using trypan blue and DAPI staining.

#### Library preparation and preprocessing

Single-nucleus multiomics was performed following the CG000338 library construction protocol from 10x Genomics. Libraries were sequenced on an Illumina Novaseq 6000 through Psomagen sequencing services. Reads were aligned to the GRCh38 human reference genome with CellRanger 8.0, and the resulting cell-by-gene count matrix was processed with the scanpy package.^104^

Cells were filtered to exclude those with more than 14% of counts mapped to mitochondrial genes, less than 200 genes, or more than 30000 unique molecular identifiers (UMIs). Cells from individual datasets were merged, normalized to total read counts, and log transformed with the percentage of mitochondrial counts and ribosomal counts used as variables for the regress_out function. Count matrixes were scaled by depth and log normalized. Nonnegative Matrix Factorization (NMF, sklearn, n_components = 20) was used for dimensionality reduction. n_components for NMF was selected based on a PCA of the data.^130^ Leiden clusters were calculated and UMAP coordinates were generated with the leiden and umap tools. Leiden clusters representing non-retinal cell types, including retinal pigmented epithelium (RPE) and brain and spinal cord-like cells (BSLCs), were manually annotated by their high expression of marker genes prior to downstream analyses.^93,96^

#### Cell type annotation and transfer learning

To call retinal cell types, we trained a random forest classifier on a reference snRNA-seq dataset of human fetal retinas.^95^ The top 50 differentially expressed genes between major retinal cell types in the fetal dataset were selected using scanpy’s rank_genes_groups tool and used to train the classifier (sklearn RandomForestClassifier, n_estimators = 200, max_features = None). The organoid dataset was subset to these genes for classification and organoid cells were assigned their highest probability cell type by the trained classifier. For organoid cells with low log likelihood, probability scores or BSLC/RPE-like expression patterns, previous manual annotations were used. Annotated non-retinal cells were removed from downstream analyses.

To validate the retinal cell type annotation by the classifier, NMF (n_components = 40) was conducted on the top 3000 highly variable genes in the reference fetal dataset. To visualize organoid cells in the same space as fetal cells in the reference dataset, fetal and organoid cells were projected onto these NMF components using scProject’s Elastic_Net tool (alpha = 0.005, L1 = 0.005).^91^

To visualize annotated organoid cells only, we subset the count matrix of the organoid dataset to a collection of the top 50 differentially expressed genes from each major cell type in the fetal reference dataset and 43 cell cycle-associated genes. NMF (n_components = 20) was conducted on the subset matrix for dimensionality reduction, and UMAP coordinates of reprojected organoid cells were generated with the umap tool.

Percent abundances of annotated organoid cell types at each timepoint were calculated by a custom script (SZ_percabun_annotated.py), and significance was assessed by chi-squared tests for proportions with Benjamini-Hochberg, multiple testing corrections.

#### Pseudotime analysis of RGCs

To quantify developmental progression along a cell fate trajectory, RGCs from the fetal and organoid datasets were subset. Organoid RGCs with low confidence scores from the classifier were excluded from the analysis to ensure rigor. Organoid RGCs were projected onto a fetal RGC UMAP using the same transfer learning pipeline described above. Fetal and organoid RGCs were assigned a pseudotime value using the scFates package’s tree tool, with fetal RGC precursors being selected as the root.^98^ To assess for significant changes in pseudotime progression in organoid RGCs, we used a permutation test. Genes associated with early, mid and late RGC fate were identified using scFates test_association tool. 5 clusters of pseudotemporally associated genes were identified, with cluster 2 and 3 corresponding to the mid-late and late maturation stages of RGCs, respectively. To evaluate maturation of RGCs by aggregate gene expression, genes from clusters 2 and 3 were used as targets for scanpy’s score_genes function to generate an RGC maturation score (Table S1).^104^ To assess for significant changes in maturity score between timepoints, permutation tests were used with Benjamini-Hochberg multiple testing correction.

### QUANTIFICATION AND STATISTICAL ANALYSIS

Manual counting and semi-automated quantification in this study were conducted using the Cell Counter and Analyze Particles function panels in ImageJ. For human fetal data, *n* = 2–3 human fetal eye samples per timepoint were quantified (*n* = 3 retinas for PCW 9, *n* = 2 retinas for PCW 13, 16 and 20). For each fetal eye, 3 interleaved sections within 100 μm to each other from a series of parasagittal sections around the central parts of the eye were quantified and averaged for a more accurate representation of quantitative measurement. Data are presented as stacked boxplots or mean ± standard deviation (SD) (Figures S1E-S1H).

For regionalized cell density quantification along the vertical meridian, images of each retinal section were split into non-overlapping 2 mm segments based on their spatial distribution along the dorsal-ventral axis. Cell densities of each segment were individually quantified. Measurements of cell densities were plotted with the central segment centered. For peak detection, a parametric hypothesis test for unimodality using quadratic regression was conducted for regional cell densities at each timepoint and a peak was called when *p* < 0.05 (Figures S1I-S1K).

For organoid data, *n* = 3–5 organoids from the same genetic background or experimental treatments per timepoint were quantified. Organoids with non-retinal regions larger than 70% of the section area were removed from analysis and assumed not to have properly differentiated. For each organoid, 3 interleaved sections with 50 μm intervals from each other were quantified and averaged for a more accurate representation of quantitative measurement. SEM was calculated for organoids with the same genotype/group, differentiated from the same batch, and collected at the same timepoint. Data are presented as boxplots or mean ± SEM with individual data points included.

For compartmentalized cell density measurement for organoid data, boundaries between retinal layers and the core on each section were manually outlined based on the enrichment of pyknotic cells by DAPI and/or PI staining. The areas of retinal layers or the core were quantified using the Measure functions in ImageJ based on the regional outlines. Apoptotic cell densities were calculated by dividing the number of apoptotic cells in retinal layers by the area of retinal layers. Necrotic cell densities were calculated by dividing the number of necrotic cells in the core by the area of the core. SNCG^+^ RBPMS^+^ cell densities were calculated by dividing the number of SNCG^+^ RBPMS^+^ cells in retinal layers by the area of retinal layers. Other cell density measurements were calculated by dividing the number of labeled cells by the area of the entire OV sections.

For retinal layer thickness measurement for organoid data, boundaries between different retinal layers were manually outlined based on the enrichment of DAPI signals and/or typical ONL- (e.g., CRX), or INL-/GCL-enriched cell type markers (e.g., TFAP2a, SNCG). The thickness of each layer was calculated and averaged by the vertical ranges with positive layer-specific signals at 8 different positions along the perimeter of OVs.

All statistical tests of the cell density and proportion data were performed using GraphPad Prism software (GraphPad Prism software v.10.4.1). To compare the differences of apoptotic or necrotic cell density or proportion, OV size, retinal layer thickness, and the proportion of core area between control and *BAX/BAK dKO* organoids at different timepoints, we used two-way ANOVA followed by Sidá k’s post hoc test (Figures 2C, 2D, 2G, 2J, and S5T-S5V). To compare the differences of cell density, proportion, or retinal layer thickness between two different genotypes or experimental groups at individual timepoints, we used unpaired two-tailed Student’s *t* test (Figures 3N, 3O, 4D, 4L-4M, S5Z, S6L, S6O, S7D, and S7E) or unpaired two-tailed Welch’s *t* test (Figure 3P). Statistical differences were considered significant when *p*-values were less than 0.05. For *p* value reported, *p < 0.05, **p < 0.01, ***p < 0.001, ****p < 0.0001, ns = not significant.

## Supplementary Material

1

2

SUPPLEMENTAL INFORMATION

Supplemental information can be found online at https://doi.org/10.1016/j.celrep.2026.117270.

## Figures and Tables

**Table:** KEY RESOURCES TABLE

REAGENT or RESOURCE	SOURCE	IDENTIFIER
Antibodies		
Donkey anti-Rabbit IgG (H + L) Secondary Antibody, AF488	Thermo Fisher Scientific	Cat #A21206; RRID: AB_253572
Donkey anti-Mouse IgG (H + L) Secondary Antibody, AF488	Thermo Fisher Scientific	Cat #A21202; RRID: AB_141607
Donkey anti-Sheep IgG (H + L) Secondary Antibody, AF488	Thermo Fisher Scientific	Cat #A11015; RRID: AB_2534082
Donkey anti-Goat IgG (H + L) Secondary Antibody, AF488	Thermo Fisher Scientific	Cat #A11055; RRID: AB_2534102
Donkey anti-Chick IgG (H + L) Secondary Antibody, AF488	Thermo Fisher Scientific	Cat #A11039; RRID: AB_2534096
Donkey anti-Rabbit IgG (H + L) Secondary Antibody, AF555	Thermo Fisher Scientific	Cat #A31572; RRID: AB_162543
Donkey anti-Rat IgG (H + L) Secondary Antibody, AF555	Thermo Fisher Scientific	Cat #A21434; RRID: AB_2535855
Donkey anti-Rabbit IgG (H + L) Secondary Antibody, AF647	Thermo Fisher Scientific	Cat #A31573; RRID: AB_2536183
Donkey anti-Mouse IgG (H + L) Secondary Antibody, AF647	Thermo Fisher Scientific	Cat #A31571; RRID: AB_162542
Goat anti-Guinea Pig IgG (H + L) Secondary Antibody, AF647	Thermo Fisher Scientific	Cat #A21450; RRID: AB_2535867
Donkey anti-Sheep IgG (H + L) Secondary Antibody, AF647	Thermo Fisher Scientific	Cat #A21448; RRID: AB_10374882
Rabbit polyclonal anti-active Caspase 3, 1:500	Cell Signaling	Cat #9661; RRID: AB_2341188
Rabbit polyclonal anti-TFAP2A, 1:200	Abcam	Cat #ab108311; RRID: AB_10861200
Goat polyclonal anti-CHAT, 1:100	Millipore	Cat #AB144P; RRID: AB_2313845
Mouse monoclonal anti-CRX, 1:100	Sigma Aldrich	Cat #WH000146M2; RRID: AB_1840990
Sheep polyclonal anti-GFP, 1:500	BioRad	Cat #4745-1051; RRID: AB_619712
Mouse monoclonal anti-ISL1/2, 1:100	DSHB	Cat #39.4D5; RRID: AB_2314683
Mouse monoclonal anti-Ki67, 1:500	BD Biosciences	Cat #550609; RRID: AB_393778
Rabbit polyclonal anti-OPN1LW, 1:200	Sigma Aldrich	Cat #AB5405; RRID: AB_177456
Chick polyclonal anti-OPN1SW, 1:200	Gift from J. Nathan lab	N/A
Mouse monoclonal anti-PAX6, 1:200	Invitrogen	Cat #MA1109; RRID: AB_2536820
Mouse monoclonal anti-POU4F1/BRN3A, 1:100	Santa Cruz	Cat #sc-8429; RRID: AB_626765
Rabbit polyclonal anti-PROX1, 1:1000	Millipore	Cat #AB5475; RRID: AB_177485
Guinea Pig polyclonal anti-RBPMS, 1:500	Invitrogen	Cat #PA5-119676; RRID: AB_2913249
Rabbit polyclonal anti-RBPMS, 1:400	Phosphosolutions	Cat #1830-RBPMS; RRID: AB_2492225
Rat polyclonal anti-RFP, 1:800	Chromotek	Cat #5f8; RRID: AB_2336064
Mouse monoclonal anti-SNCG, 1:500	Abnova	Cat #H00006623-M01; RRID: AB_464249
Sheep polyclonal anti-VSX2/CHX10, 1:300	Exalpha	Cat #X1180P; RRID: AB_2314191
Biological samples		
Human fetal eye tissue	Developmental Origin of Health and Disease biore pository at Mount Sinai	https://icahn.mssm.edu/about/departments/regenerative-biology
Chemicals, peptides, and recombinant proteins		
mTeSR^™^1	Stem Cell Technologies	Cat #85857
Matrigel-GFR^™^	BD Biosciences	Cat #354230
Accutase	Sigma	Cat #SCR005
Blebbistatin	Sigma	Cat #B0560
Insulin	Roche	Cat #11376497001
holo-transferrin	Sigma	Cat #T0665
L-ascorbic acid	Sigma	Cat #A8960
sodium selenite	Sigma	Cat #S5261
B27 supplement minus Vitamin A (50X)	Thermo Fisher Scientific	Cat #12587010
Glutamax	Thermo Fisher Scientific	Cat #35050061
NEAA	Thermo Fisher Scientific	Cat #11140050
Sodium Pyruvate	Thermo Fisher Scientific	Cat #11360070
F12	Thermo Fisher Scientific	Cat #11765062
B27 supplement (50X)	Thermo Fisher Scientific	Cat #17504044
heat-inactivated FBS	Thermo Fisher Scientific	Cat #16140071
taurine	Sigma	Cat #T-8691
NaCl	Sigma	Cat #S3014
DMEM	Thermo Fisher Scientific	Cat #11885084
all-trans retinoic acid (ATRA)	Sigma	Cat #R2625
Gamma-secretase inhibitor (DAPT)	EMD Millipore	Cat #565770
Formaldehyde (16%), Methanol-free	Thermo Fisher Scientific	Cat #28906
PBS	Thermo Fisher Scientific	Cat #AM9624
O.C.T	Sakura Finetek	Cat #12351753
Donkey serum	Millipore	Cat #S30-M
Triton X-100	Sigma	Cat #T9284
DAPI	Thermo Fisher Scientific	Cat #62248
Hoechst	Biotium	Cat #40046
SlowFade Gold Antifade mountant	Thermo Fisher Scientific	Cat #P36940
Propidium iodide	Sigma-Aldrich	Cat #P4170
Critical commercial assays		
MycoAlert	Lonza	Cat #LT07
Click-iT^™^ Plus TUNEL Assay	Invitrogen	Cat #C10617
Click-iT EdU Assay	Invitrogen	Cat #C10640
Deposited data		
10x Chromium snRNA-seq data	This paper	GSE305194
Original code for snRNA-seq analysis	This paper	https://github.com/BGuy2/SZ_BAX-BAKdKO/
Experimental models: Cell lines		
H7 POU4F2-P2A-tdTomato-P2A-mTHY1.2 hESC line	Prof. Donald Zack, JHMI	Sluch et al.^58^
H9 POU4F2-P2A-mNeon-H2B hESC line	Prof. Karl Wahlin, UCSD	Agarwal et al.^64^
IMR90.4 SIX6-P2A-H2B-GFP hiPSC line	Prof. Donald Zack, JHMI	Wahlin et al.^79^
GM25256 iPSC line	Prof. Vivian Gama, Vanderbilt University	Joshi et al.^87^
GM25256 BAX/BAK double knockout iPSC line	Prof. Vivian Gama, Vanderbilt University	Joshi et al.^87^
Software and algorithms		
Fiji	ImageJ	https://imagej.net/software/fiji/
Prism v10.4.1	GraphPad software	https://www.graphpad.com/
Python v3.9.6 and v11.3.4	Python Software Foundation	https://www.python.org/
CellRanger v8.0	10x Genomics	https://www.10xgenomics.com/support/software/cell-ranger/latest
scanpy v1.9.3	Wolf et al.^104^	https://scanpy.readthedocs.io
sklearn v1.7.2	Pedregosa et al.^130^	https://scikit-learn.org
scProject v1.1.4	Stein-O’Brien et al.^91^	https://github.com/gofflab/scProject
scFates v1.1.1	Faure et al.^98^	https://scfates.readthedocs.io/en/latest/

## Highlights

- Two apoptotic waves affect RPCs and RGCs in human retinas and retinal organoids
- Retinal organoids undergo a distinct wave of necrosis during development
- Blocking apoptosis extends RGC lifespan and enhances RGC survival
- Apoptosis inhibition delays RGC neurogenesis and maturation
