# Supplementary material for: Blocking apoptosis promotes survival and alters developmental dynamics of human retinal ganglion cells in retinal organoids
Source: Cell Rep. Author manuscript; Available in PMC 2026 May 18. (PMC13181862; doi:10.1016/j.celrep.2026.117270)
Supplement: 1 [file NIHMS2170850-supplement-1.pdf]

Cell Reports, Volume *45*

Supplemental information

Blocking apoptosis promotes survival and alters developmental dynamics of human retinal ganglion cells in retinal organoids

Jingliang Simon Zhang (张景亮), Brian Guy, Clayton P. Santiago, Caterina Tiozzo, Meghana Sreenath, Ya-Wen Chen, Seth Blackshaw, and Robert J. Johnston Jr.

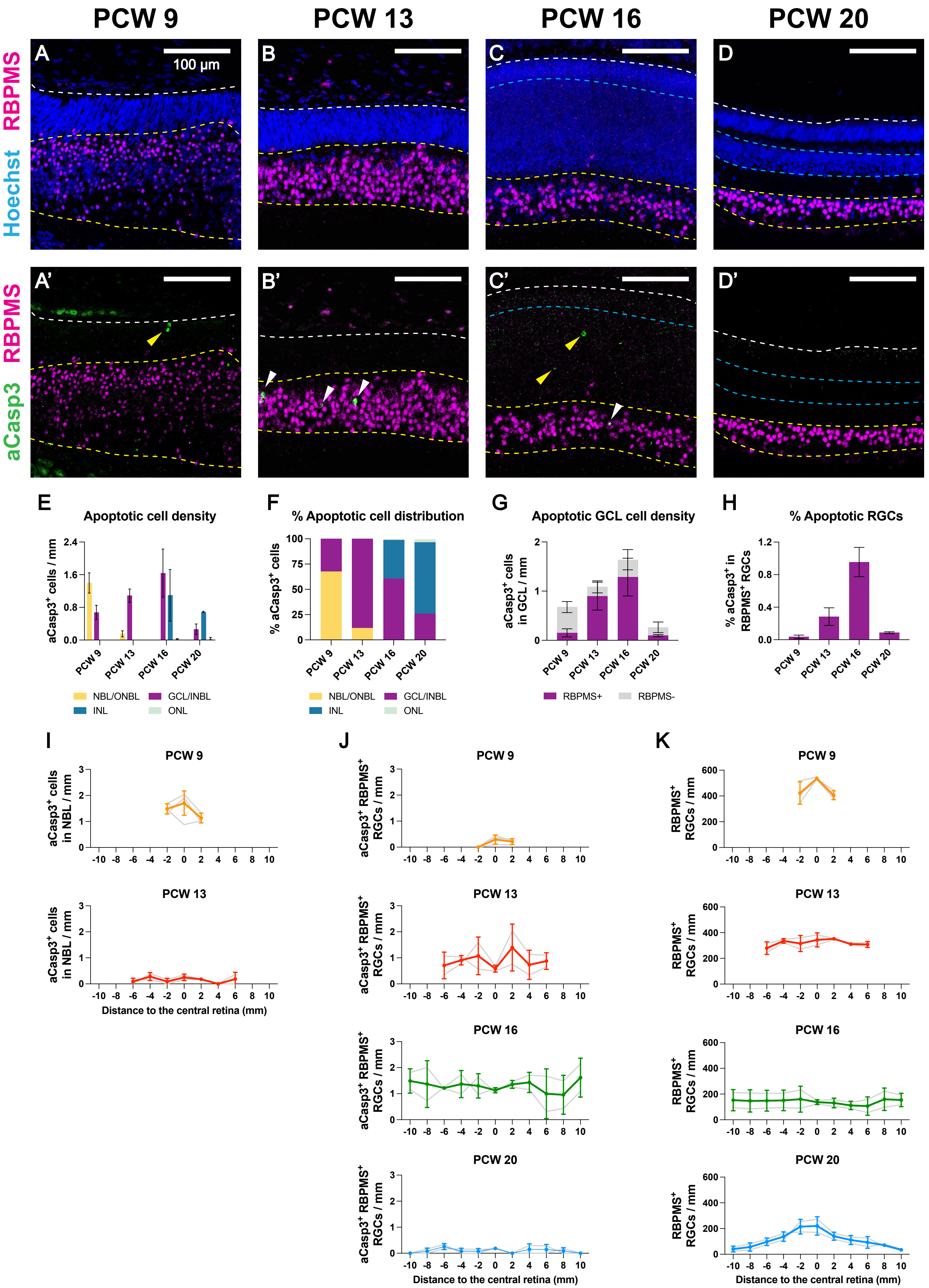

Supplementary Figure 1

Figure S1. Two waves of apoptosis in developing human retinas

(A-D) Immunofluorescent staining of human fetal retina sections at post-conception week 9 (A), 13 (B), 16 (C) and 20 (D). The apical boundary of the neural retina (white dashed line) and the boundary of the INL (cyan dashed line) are outlined based on DAPI staining. The boundary of the GCL (yellow dashed line) is outlined based on RBPMS staining (magenta) in the GCL. White arrowheads indicate aCasp3$^+$ RBPMS$^+$ cells (apoptotic RGCs). Yellow arrowheads indicate aCasp3$^+$ RBPMS$^-$ cells (apoptotic non-RGCs). Scale bar, 100 μm.

(E-F) Density (E) and laminar distribution (F) of apoptotic cells in layers of human fetal retinas at PCW 9, 13, 16 and 20. At PCW 9 and 13, the pseudostratified retinas are comprised of the NBL (or outer neuroblastic layer, ONBL) and the laminating GCL (or inner neuroblastic layer, INBL). At PCW 16 and 20, the fetal retinas are comprised of the ONL, INL and GCL. Data are represented as mean ± SD. N = 3 for PCW 9, N = 2 for PCW 13, N = 2 for PCW 16, N = 2 for PCW 20.

(G) Density of apoptotic RBPMS$^+$ and RBPMS$^-$ cells in the GCL of human fetal retinas at PCW 9, 13, 16 and 20. Data are represented as mean ± SD. N = 3 for PCW 9, N = 2 for PCW 13, N = 2 for PCW 16, N = 2 for PCW 20.

(H) Proportion of apoptotic RBPMS$^+$ RGCs of all RBPMS$^+$ RGCs in human fetal retinas at PCW 9, 13, 16 and 20. Data are represented as mean ± SD. N = 3 for PCW 9, N = 2 for PCW 13, N = 2 for PCW 16, N = 2 for PCW 20.

(I) Regional density of aCasp3$^+$ cells in the NBL along the vertical meridian of human fetal retinas. Colored lines represent mean ± SD. Grey lines represent individual samples. N = 3 for PCW 9, N = 2 for PCW 13, N = 2 for PCW 16, N = 2 for PCW 20.

(J) Regional density of aCasp3$^+$ RBPMS$^+$ RGCs along the vertical meridian of human fetal retinas. Colored lines represent mean ± SD. Grey lines represent individual samples. N = 3 for PCW 9, N = 2 for PCW 13, N = 2 for PCW 16, N = 2 for PCW 20.

(K) Regional density of RBPMS$^+$ RGCs along the vertical meridian of human fetal retinas. Colored

lines represent mean ± SD. Grey lines represent individual samples. N = 3 for PCW 9, N = 2 for PCW 13, N = 2 for PCW 16, N = 2 for PCW 20.

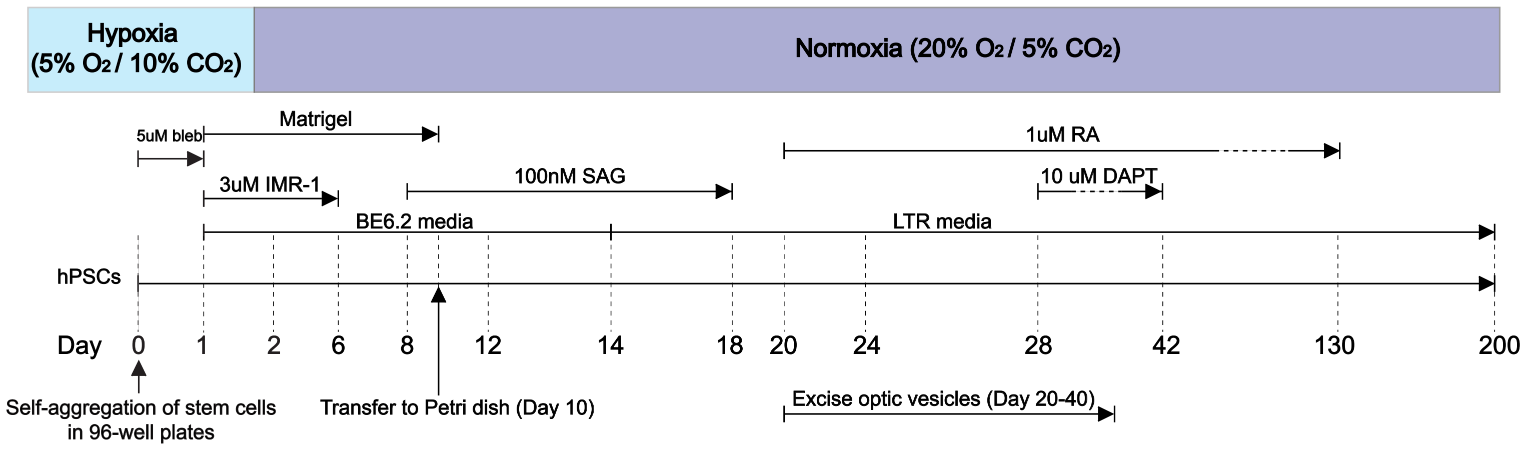

Supplementary Figure 2

Figure S2. Gravity aggregation protocol of human retinal organoid differentiation

Gravity aggregation protocol adapted from previous studies.[59,60]

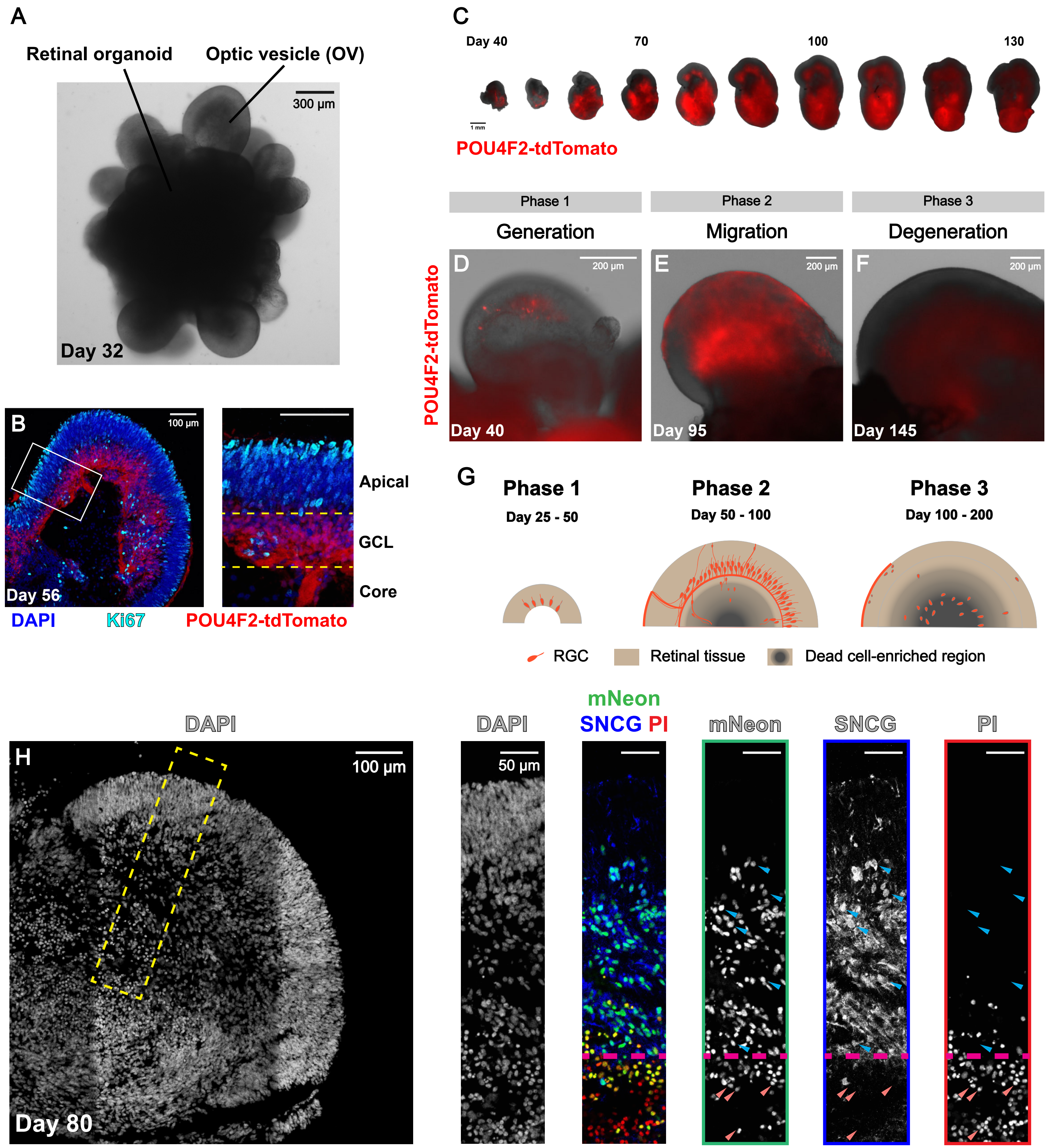

Supplementary Figure 3

Figure S3. RGC migration and loss shown by RGC-specific reporters in human retinal organoids

(A) Differential interference contrast (DIC) image of a day 32 retinal organoid with OVs. Scale bar, 300 μm.

(B) OV with lamination of the NBL, GCL, and the core. (Inset) The boundaries of the NBL, GCL and the core (yellow dashed line) are indicated by $Ki67^+$ RPCs (cyan) in the NBL and POU4F2-$tdTomato^+$ RGCs (red) in the GCL. Scale bar, 100 μm.

(C) Overlaying DIC and red fluorescent images of a single H7 *POU4F2-tdTomato* retinal organoid from day 40 to day 130. Scale bar, 1mm.

(D-F) Overlaying DIC and fluorescent images of Phase 1 (D), Phase 2 (E), and Phase 3 (F) organoids. Scale bar, 200 μm.

(G) Schematic of three phases of RGC development and loss in human retinal organoids.

(H) mNeon labeled both live and dead RGCs in human retinal organoids. A region of interest is outlined and shown in the insets (yellow dashed line). (Insets) Live $mNeon^+$ RGCs ($SNCG^+$ PI^- $mNeon^+$, blue arrowhead) express the RGC-specific marker SNCG and are predominantly located in the retinal layers. Dying and dead $mNeon^+$ RGCs ($SNCG^-$ PI^+ $mNeon^+$, orange arrowhead), labeled by propidium iodide (PI), are mostly located in the core. The boundary between the retinal layers and the core of OVs (magenta dashed line) is outlined based on the distribution of pyknotic nuclei and PI staining. Scale bar, 100 μm on the left and 50 μm on the right.

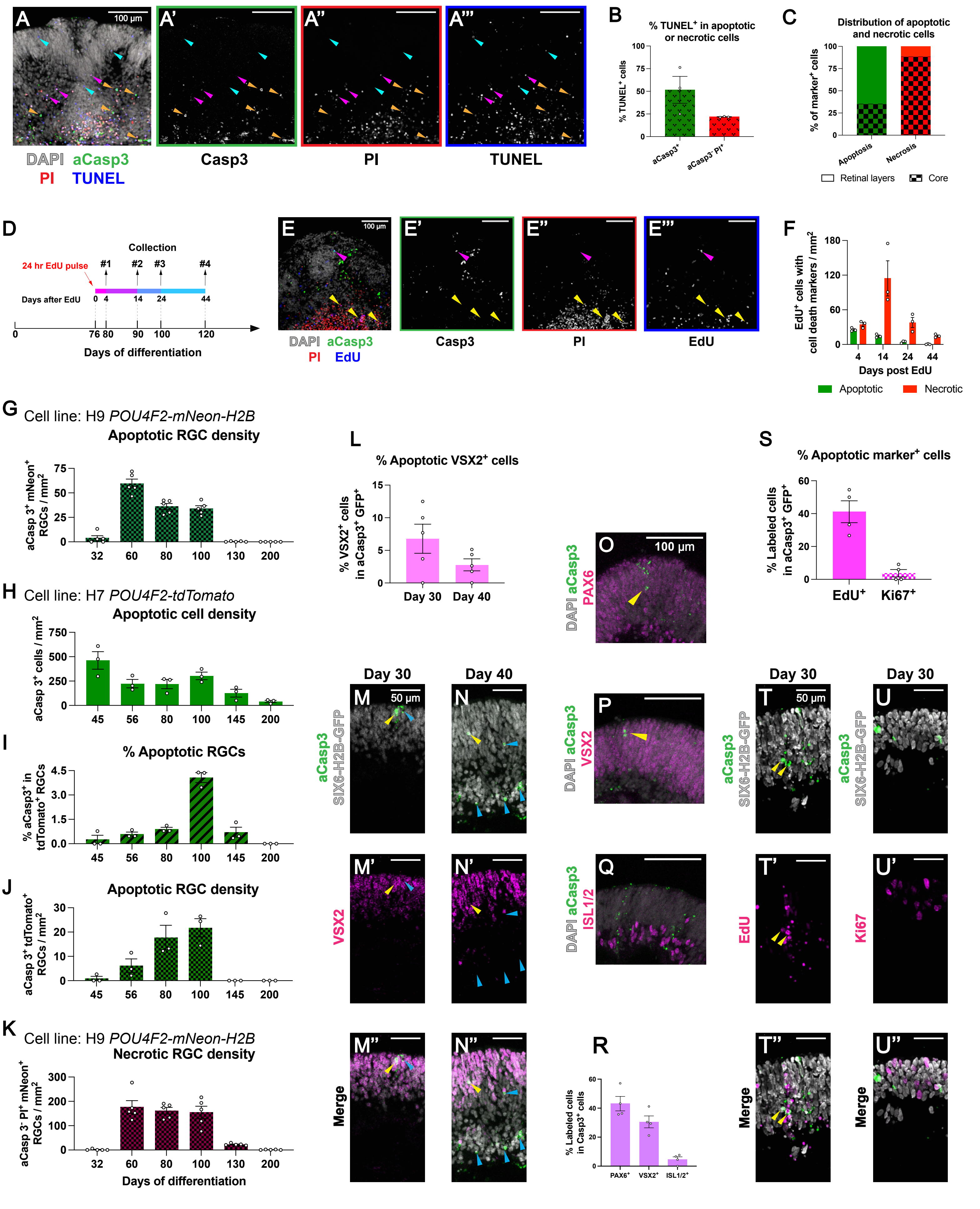

Supplementary Figure 4

Figure S4. Measurements of apoptosis and necrosis in human retinal organoids

(A) Co-detection of dead cells with aCasp3, PI, and TUNEL staining in day 60 organoids derived from GM25256 hiPSCs. Orange arrowheads indicate $TUNEL^+$ $aCasp3^+$ cells ($TUNEL^+$ apoptotic cells). Magenta arrowheads indicate $TUNEL^+$ $aCasp3^-$ PI^+ cells ($TUNEL^+$ necrotic cells). Cyan arrowheads indicate $TUNEL^+$ $aCasp3^-$ PI^- cells ($TUNEL^+$ cells without apoptotic or necrotic markers). Scale bar, 100 μm.

(B) % $TUNEL^+$ cells of apoptotic or necrotic cells in day 60 retinal organoids. Data are represented as mean ± SEM. N = 3 organoids.

(C) Distribution of apoptotic and necrotic cells in retinal layers (solid) and the core (checkerboard) of day 60 organoids. N = 3 organoids.

(**D**) Experimental design of EdU labeling of early apoptotic cells. EdU was pulsed for 24 hours on day 76. EdU-treated organoids were collected 4, 14, 24 and 44 days later.

(**E**) Co-detection of cell death markers in EdU-labeled cells. Magenta arrowheads indicate apoptotic EdU-labeled cells ($aCasp3^+$ EdU^+). Yellow arrowheads indicate necrotic EdU-labeled cells ($aCasp3^-$ PI^+ EdU^+). Scale bar, 100 μm.

(**F**) Density of apoptotic and necrotic EdU^+ cells in GM25256 retinal organoids. Data are represented as mean ± SEM. N = 3 organoids per timepoint.

(**G**) Density of $aCasp3^+$ $mNeon^+$ apoptotic RGCs in retinal layers of H9 *POU4F2-mNeon-H2B* retinal organoids. Data are represented as mean ± SEM. N = 5 organoids per timepoint.

(H-J) Density of apoptotic cells (H), % apoptotic $tdTomato^+$ RGCs (I), and density of apoptotic $tdTomato^+$ RGCs (J) in H7 *POU4F2-tdTomato* retinal organoids. Data are represented as mean ± SEM. N = 3 organoids per timepoint.

(K) Density of $aCasp3^-$ PI^+ $mNeon^+$ necrotic RGCs in the core of H9 *POU4F2-mNeon-H2B* OVs. Data are represented as mean ± SEM. N = 5 organoids per timepoint.

(L-N) $VSX2^+$ RPCs in the $aCasp3^+$ GFP^+ population during the early wave of apoptosis. (L) % $VSX2^+$ cells of all $aCasp3^+$ GFP^+ cells on days 30 and 40. Data are represented as mean ± SEM.

N = 5 organoids per timepoint. (M-N) VSX2$^+$ (yellow arrowheads) and VSX2$^-$ (blue arrowheads) cells among the aCasp3$^+$ GFP$^+$ cells in day 30 (M) and day 40 (N) organoids. Scale bar, 50 μm.

(O-R) Identification of cell types of aCasp3$^+$ population in day 30 *H9 POU4F2-mNeon-H2B* organoids. (O-Q) aCasp3$^+$ cells that are PAX6$^+$ (O), VSX2$^+$ (P), ISL1/2$^+$ (Q). Yellow arrowheads indicate double positive cells. Scale bar, 100 μm. (R) % marker positive cells in the aCasp3$^+$ population. N = 4 organoids. Data are represented as mean ± SEM.

(S-U) Identification of cell types of the aCasp3$^+$ GFP$^+$ population during the early wave of apoptosis. (S) % aCasp3$^+$ GFP$^+$ labeled by EdU$^+$ or Ki67$^+$ of all aCasp3$^+$ GFP$^+$ cells. Data are represented as mean ± SEM. N = 4 organoids. (T-U) EdU$^+$ (yellow arrowhead, T) or Ki67$^+$ cells (U) that are aCasp3$^+$ GFP$^+$ in day 30 organoids. Scale bar, 50 μm.

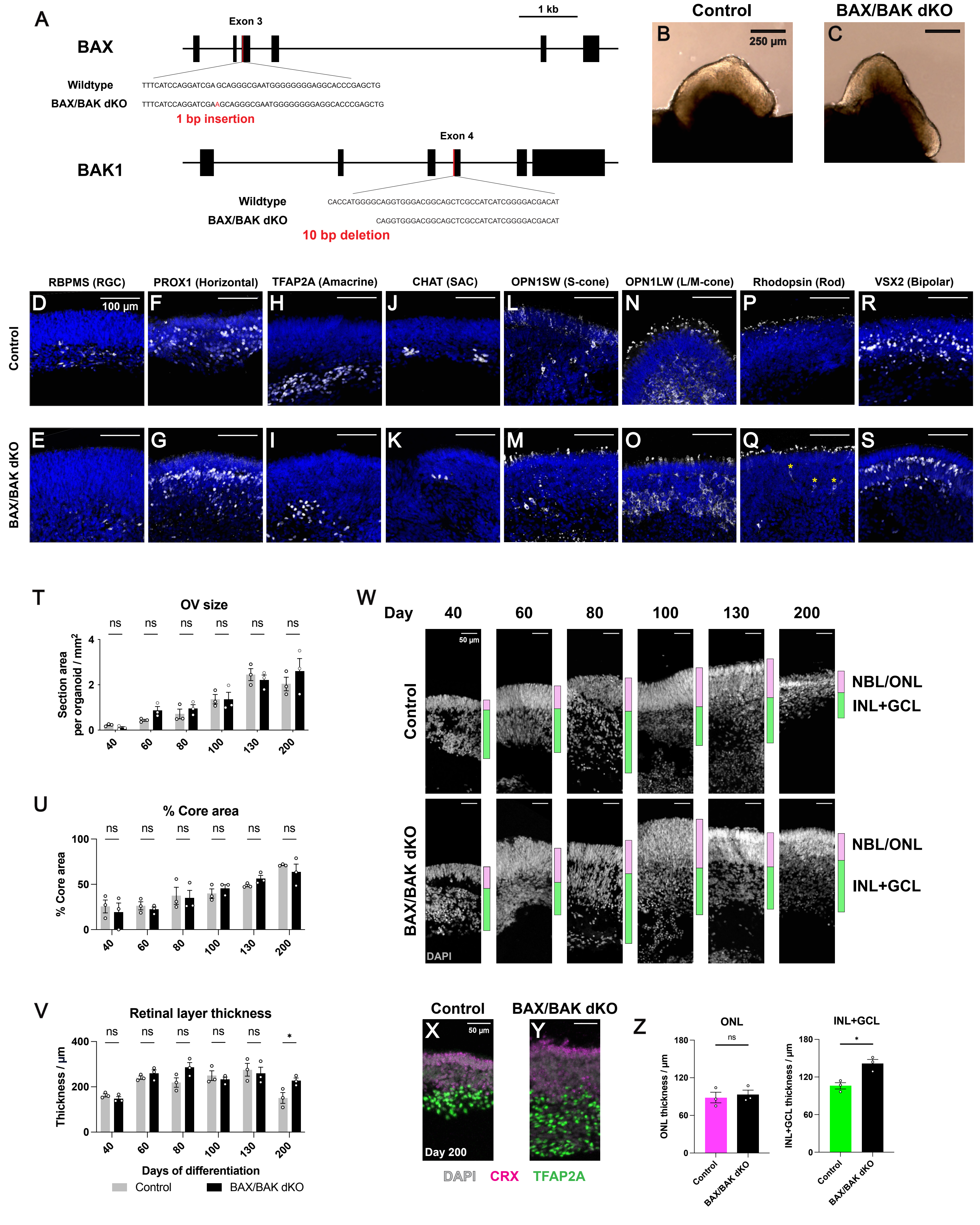

Supplementary Figure 5

Figure S5. Development of human control and *BAX/BAK dKO* retinal organoids

(A) Schematic of mutations generated in *BAX* and *BAK1* (*BAK*) loci. Black bars indicate exons. Black lines between the bars indicate introns. Red bars indicate the location of mutations.

(B-C) Brightfield images of OVs of day 25 control (B) and *BAX/BAK dKO* (C) retinal organoids. Scale bar, 250 μm.

(D-S) Major neuronal types are generated in control and *BAX/BAK dKO* retinal organoids, including RGCs (D-E, RBPMS^{+}, day 100), horizontal cells (F-G, PROX1^{+}, day 200), amacrine cells (H-I, TFAP2A^{+}, day 200), starburst amacrines/SAC (J-K, CHAT^{+}, day 200), S-cone photoreceptors (L-M, OPN1SW^{+}, day 200), L/M-cone photoreceptors (N-O, OPN1LW^{+}, day 200), rod photoreceptors (P-Q, Rhodopsin^{+}, day 200), and bipolar cells (R-S, VSX2^{+}, day 200). Yellow asterisks indicate ectopic Rhodopsin^{+} rods in *BAX/BAK dKO* organoids. Scale bar, 100 μm.

(T-V) Quantifications of OV size (T), % of core area (U), and thickness of retinal layers (V) in control and *BAX/BAK dKO* organoids. Data are represented as mean ± SEM. N = 3 organoids per timepoint. Two-way ANOVA followed by Šidák's post hoc test, * $p < 0.05$, ns = not significant.

(**W**) Lamination of control and *BAX/BAK dKO* organoids during differentiation. Magenta bars indicate the NBL or ONL. Green bars indicate the INL+GCL. Scale bar, 50 μm.

(**X-Y**) Lamination of day 200 control (X) and *BAX/BAK dKO* organoids (Y). CRX (green) marks the region of the ONL, and TFAP2a (magenta) marks the region of the INL+GCL. Scale bar, 50 μm.

(**Z**) Quantification of ONL (left) and INL+GCL (right) thickness in day 200 control and *BAX/BAK dKO* organoids. Data are represented as mean ± SEM. N = 3 organoids per genotype. Unpaired two-tailed student's t-test, * $p < 0.05$, ns = not significant.

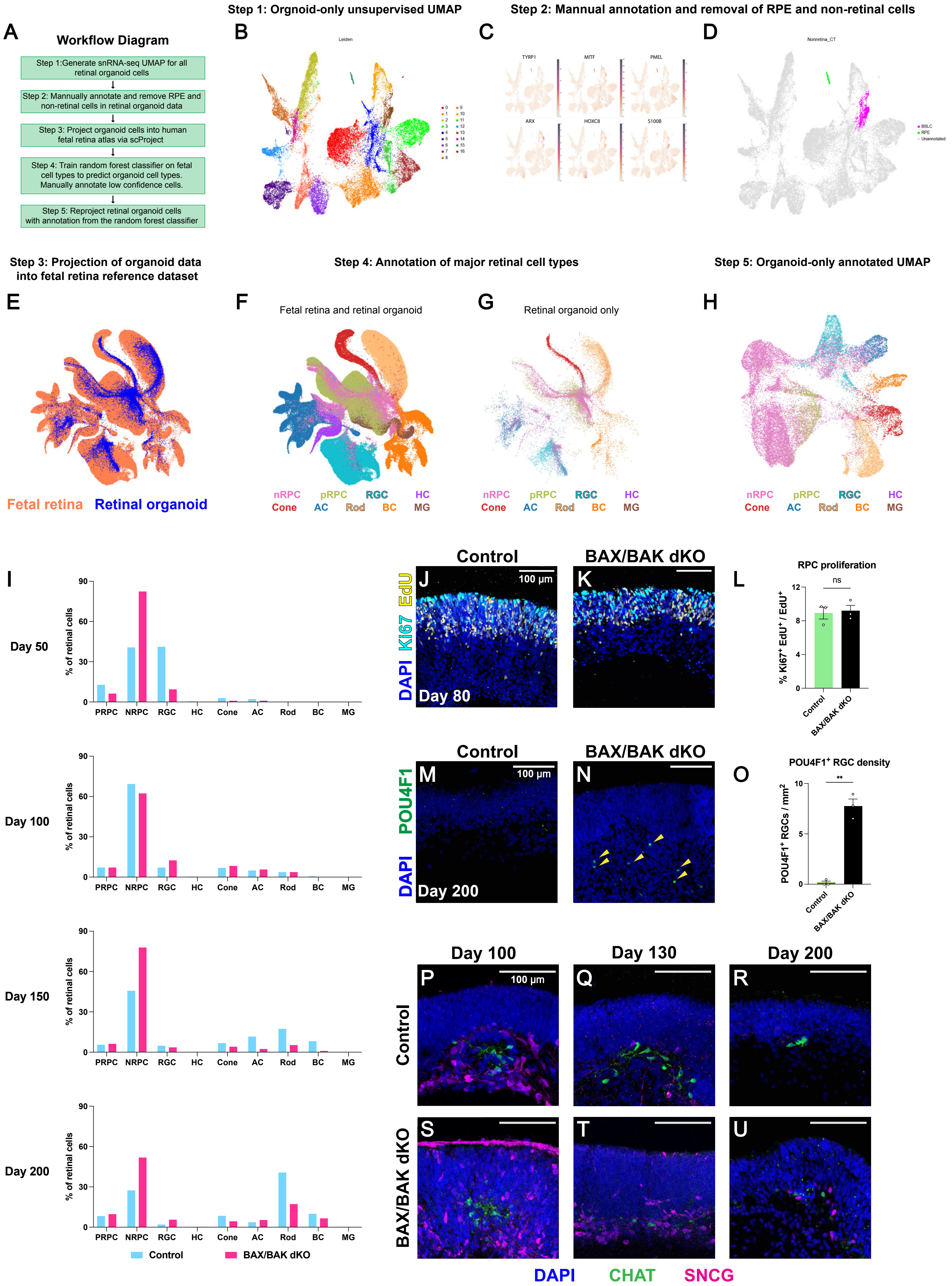

Supplementary Figure 6

Figure S6. Cell type composition and RGC-SAC interactions of control and *BAX/BAK dKO* organoids

(A) Diagram of major cell class annotation workflow. In steps 1-2, organoid-only data underwent dimension reduction and were projected onto UMAPs. RPE and non-retinal cells (brain and spinal cord-like cells, BSLCs) were manually annotated by known markers and removed from downstream analysis. In steps 3-4, organoid data were mapped to the reference fetal UMAP with scvi-tools and annotated by a random forest classifier trained on the fetal data. In step 5, annotated organoid-only data were reprojected.

(B) Organoid-only data were projected and clustered onto a UMAP in an unsupervised manner.

(C-D) RPE and non-retinal cells were manually annotated based on expression of known marker genes. (C) Expression of RPE and BSLC marker genes (*TYRP1*, *MITF*, *PMEL*, *ARX*, *HOXC8*, *S100B*). (D) RPE (green) and BSLC clusters (magenta) are highlighted in the unsupervised UMAP from step 1.

(E) UMAP of integrated fetal retina and retinal organoid data from step 2. UMAP is colored by sample sources (fetal = orange, organoid = blue).

(F-G) UMAPs of inferred major class annotation for development data from step 3. Integrated (F) and organoid-only UMAP (G) with annotation is shown.

(H) UMAP of organoid-only data from step 4. At this step, fetal retina data were removed, and organoid-only data are reprojected.

(I) % of retinal cell type abundance at each collection timepoint. Blue = control; red = *BAX/BAK dKO*. Difference in the proportions for cone photoreceptors, horizontal cells, amacrine cells, and Müller glia were minimal. Proportions of rod photoreceptors and bipolar cells were decreased in *BAX/BAK dKO* organoids on days 150 and 200, suggesting that blocking apoptosis impedes their neurogenesis.

(J-K) $Ki67^{+}$ EdU^{+} pRPCs in day 80 control (J) and *BAX/BAK dKO* (K) retinal organoids. EdU treatments for 24 hours were performed on day 76, and EdU-treated organoids were collected 4

days after each treatment. Scale bar, 100 μm.

(L) % Ki67$^+$ EdU$^+$ cells of all EdU$^+$ cells in day 80 control and *BAX/BAK dKO* organoids. Data are represented as mean ± SEM. N = 3 organoids. Unpaired two-tailed student's t-test, ns = not significant.

(M-N) POU4F1$^+$ RGCs (green, yellow arrowhead) in day 200 control (M) and *BAX/BAK dKO* (N) organoids. Scale bar, 100 μm.

(O) Density of POU4F1$^+$ RGCs in day 200 control and *BAX/BAK dKO* organoids. Data are represented as mean ± SEM. N = 3 organoids. Unpaired two-tailed Welch's t-test, ** $p < 0.01$.

(P-U) RGC-SAC interactions in control and *BAX/BAK dKO* organoids on days 100 (P, S), 130 (Q, T), and 200 (R, U). SACs are labeled by CHAT (green), and RGCs are labeled by SNCG (magenta). Scale bar, 100 μm.

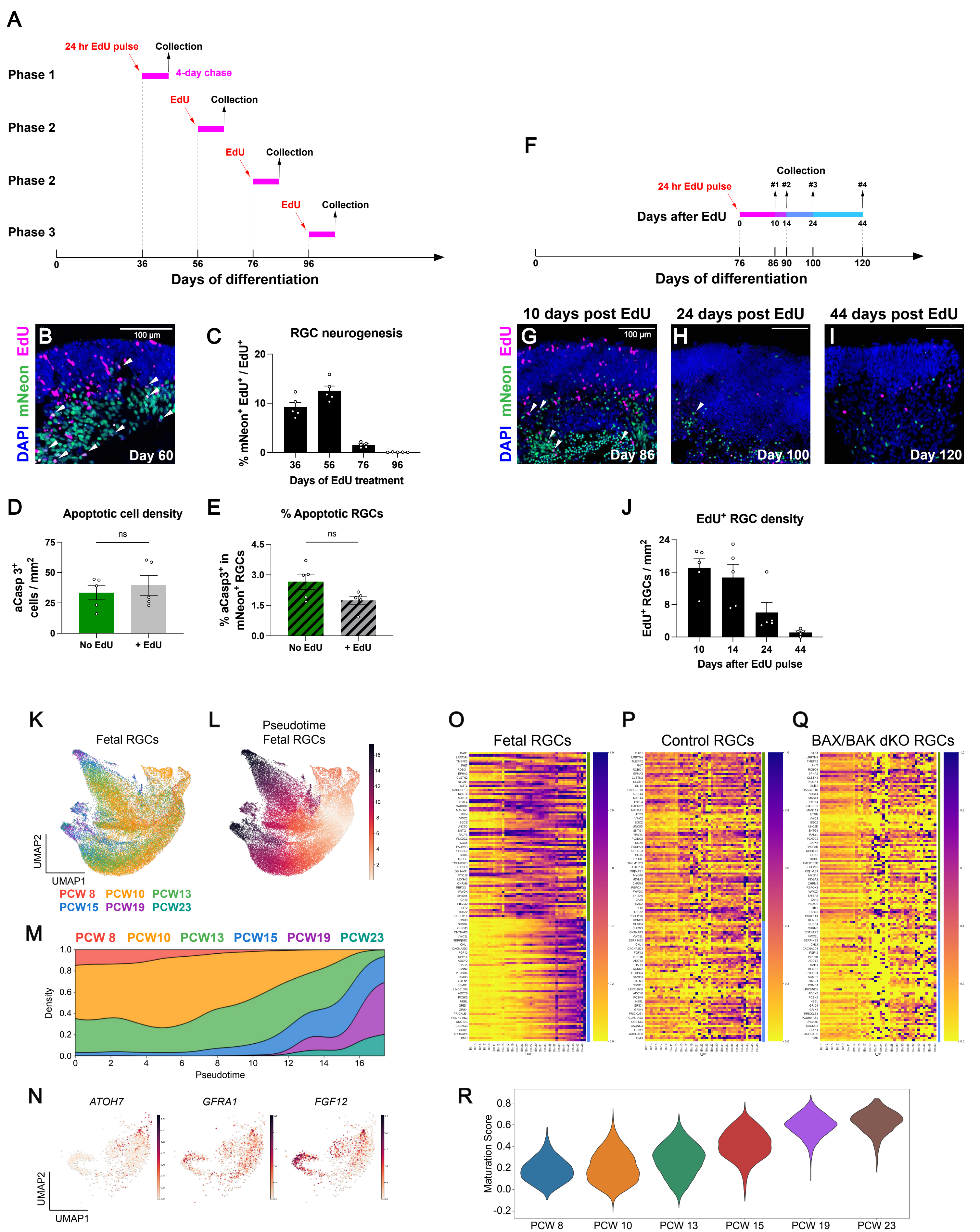

Supplementary Figure 7

Figure S7. RGC neurogenesis, survival, and maturation in human control and *BAX/BAK dKO* retinal organoids

(A) Experimental design of RGC neurogenesis measurement by EdU labeling. EdU was pulsed for 24 hours on days 36, 56, 76 or 96. EdU-treated organoids were collected 4 days after each treatment.

(B) EdU^+ $mNeon^+$ RGCs (white arrowhead) mark the RGC population born during the EdU treatment. Scale bar, 100 µm.

(C) % newborn EdU^+ $mNeon^+$ RGCs of all EdU^+ cells. Data are represented as mean ± SEM. N = 5 organoids per timepoint.

(**D-E**) Density of apoptotic cells (D) and % apoptotic RGCs of all $mNeon^+$ RGCs (E) in day 80 untreated (no EdU) and EdU-treated (EdU treatment at day 76) H9 *POU4F2-mNeon-H2B* retinal organoids. Data are represented as mean ± SEM. N = 5 organoids per timepoint. Unpaired two-tailed student's t-test, ns = not significant.

(F) Experimental design of RGC lifespan measurement by EdU labeling. EdU was pulsed for 24 hours on day 76. EdU-treated H9 *POU4F2-mNeon-H2b* organoids were collected 10, 14, 24 and 44 days after the treatment.

(G-I) EdU^+ $mNeon^+$ RGCs (white arrowheads) 10 (G), 24 (H) and 44 (I) days after the EdU treatment. Scale bar, 100 µm.

(J) Density of EdU^+ $mNeon^+$ RGCs after EdU treatment. Data are represented as mean ± SEM. N = 5 organoids per timepoint.

(K-L) Fetal-derived RGC UMAPs colored by fetal age (K) and pseudotime (L).

(M) Stacked density distribution of human fetal RGC data from the reference dataset along the pseudotime trajectory. Colors reflect different fetal ages of RGCs in the reference dataset.

(N) Organoid-derived RGC UMAPs of representative genes in different RGC developmental stages (*ATOH7* = early, *GFRA1* = middle, *FGF12* = late) colored by expression level.

(O-Q) Heatmaps of maturation-associated gene expression in the reference fetal (O), control

organoid (P) and *BAX/BAK dKO* organoid (Q) RGCs binned by pseudotime units. Color blocks indicate genes associated with mid-late (green) and late (blue) stages of RGC maturation.

(R) Violin plot of RGC maturation scores of human fetal RGCs in different PCWs.
